# Comprehensive virulence profiling and evolutionary analysis of specificity determinants in *Staphylococcus aureus* two-component systems

**DOI:** 10.1128/msystems.00130-24

**Published:** 2024-03-12

**Authors:** Stephen Dela Ahator, Karoline Wenzl, Kristin Hegstad, Christian S. Lentz, Mona Johannessen

**Affiliations:** 1 Research Group for Host-Microbe Interactions, Centre for New Antibacterial Strategies (CANS), Department of Medical Biology, Faculty of Health Sciences, UiT- The Arctic University of Norway, Tromsø, Norway; 2 Norwegian National Advisory Unit on Detection of Antimicrobial Resistance, Department of Microbiology and Infection Control, University Hospital of North Norway, Tromsø, Norway; Pacific Northwest National Laboratory, Richland, Washington, USA

**Keywords:** two-component system, evolution, coevolution, selection pressure, virulence, specificity determinants

## Abstract

In the *Staphylococcus aureus* genome, a set of highly conserved two-component systems (TCSs) composed of histidine kinases (HKs) and their cognate response regulators (RRs) sense and respond to environmental stimuli, which drive the adaptation of the bacteria. This study investigates the complex interplay between TCSs in *S. aureus* USA300, a predominant methicillin-resistant *S. aureus* strain, revealing shared and unique virulence regulatory pathways and genetic variations mediating signal specificity within TCSs. Using TCS-related mutants from the Nebraska Transposon Mutant Library, we analyzed the effects of inactivated TCS HKs and RRs on the production of various virulence factors, *in vitro* infection abilities, and adhesion assays. We found that the TCSs’ influence on virulence determinants was not associated with their phylogenetic relationship, indicating divergent functional evolution. Using the co-crystallized structure of the DesK-DesR from *Bacillus subtilis* and the modeled structures of the four NarL TCSs in *S. aureus*, we identified interacting residues, revealing specificity determinants and conservation within the same TCS, even from different strain backgrounds. The interacting residues were highly conserved within strains but varied between species due to selection pressures and the coevolution of cognate pairs. This study unveils the complex interplay and divergent functional evolution of TCSs, highlighting their potential for future experimental exploration of phosphotransfer between cognate and non-cognate recombinant HK and RRs.

**IMPORTANCE:**

Given the widespread conservation of two-component systems (TCSs) in bacteria and their pivotal role in regulating metabolic and virulence pathways, they present a compelling target for anti-microbial agents, especially in the face of rising multi-drug-resistant infections. Harnessing TCSs therapeutically necessitates a profound understanding of their evolutionary trajectory in signal transduction, as this underlies their unique or shared virulence regulatory pathways. Such insights are critical for effectively targeting TCS components, ensuring an optimized impact on bacterial virulence, and mitigating the risk of resistance emergence via the evolution of alternative pathways. Our research offers an in-depth exploration of virulence determinants controlled by TCSs in *S. aureus*, shedding light on the evolving specificity determinants that orchestrate interactions between their cognate pairs.

## INTRODUCTION

*Staphylococcus aureus*, a commensal yet opportunistic bacterium, colonizes the skin and mucous membranes of nearly 30% of healthy adults. This adaptable pathogen is responsible for various hospital and community-acquired infections due to its production of a diverse array of virulence factors. These factors include cell wall-anchored proteins, such as protein A, fibronectin-binding proteins, and clumping factors (1–3), and secreted exoproteins, such as nucleases, proteases, lipases, collagenases, and hemolysins (1).

Bacteria have evolved sophisticated regulatory mechanisms, such as the two-component system (TCS), which integrates virulence regulation with responses to host and environmental factors (4). A typical TCS comprises a membrane-spanning sensor histidine kinase (HK) that detects stimuli, autophosphorylates, and subsequently phosphorylates a conserved aspartate residue on the cytoplasmic response regulator (RR). This second phosphorylation event induces conformational changes in the RR, enabling specific binding to DNA motifs to enact appropriate responses. The HK typically has two conserved domains: the dimerization and histidine phosphotransfer (DHp) domain for autophosphorylation and phosphotransfer and the catalytic and ATP-binding (CA) domains (4, 5). HKs usually have an N-terminal transmembrane domain for cell membrane attachment and signal perception. Additional domains, such as PAS (Per Arnt Sim), HAMP (histidine kinase, adenyl cyclases, methyl-accepting proteins, and phosphatases), and GAF (cGMP-specific phosphodiesterases, adenylyl cyclases, and FhlA) (6), help relay signals from sensory domains to DHp and CA (6–8). RRs contain a conserved receiver domain that facilitates phosphotransfer from the cognate HK, activating the DNA-binding output domain for transcriptional regulation (9). The TCS’s fundamental architecture is highly adaptable, with variations emerging from its modular architecture, domain shuffling, and sensor domain diversification, allowing the detection of diverse stimuli (10–13).

TCSs play a significant role in bacterial evolution, with their functional importance influencing their evolutionary history. Acquired through gene duplication and horizontal gene transfer, the maintenance of these genes depends on their short-term selective advantage or functional specialization (14, 15). Coevolution of HKs and cognate RRs occurs through mutations, ensuring specificity while avoiding unwanted crosstalk (8, 16–18). Most *S. aureus* strains contain 15–17 TCSs, classified into four families: NarL, AraC, OmpR, and LytTR, based on the homology of the RR effector domain and sequences surrounding the active-site histidine sequences of the HK (5, 19, 20). Despite specialized functionality, many TCSs in *S. aureus* have overlapping regulons, which are linked to their regulation of alternative regulatory pathways and the maximal expression of virulence determinants (8).

TCSs have been proposed as potential targets for antibacterial agents due to their functionality, conservation, and distribution (21). Although several studies have analyzed the functions of individual *S. aureus* TCSs independently, the first systematic study on *S. aureus* TCS at the global scale was performed by Villanueva and coworkers (22), who first generated an *S. aureus* strain deprived of all non-essential TCS systems and complemented the constitutively active forms of all TCS-RRs (23). While this deconstructive approach has been extremely powerful in determining the regulons of all individual TCS-RRs in isolation (23), the potential crosstalk between different TCS systems and their relative contributions to bacterial virulence within the complex gene-regulatory network in non-sensory-deprived cells remain ill-defined.

Here, we are providing a complementary angle on the role of individual TCSs on virulence, evolution, and their determinants of specificity. We report the results of the systemic analysis of TCSs in the *S. aureus* USA300 genome, a predominant methicillin-resistant *S. aureus* (MRSA), and a major cause of severe community-acquired infections and antibiotic resistance. Using a comprehensive panel of strains from the Nebraska Transposon Mutant Library deficient in individual TCS components, we examined how TCSs control a panel of virulence determinants common to *S. aureus* and clustered them accordingly. For a selection of TCS components and assay systems, these data were confirmed using in-*trans* gene complementation. Furthermore, the evolutionary stability of the TCSs from the genomes of *S. aureus* was determined to verify the conservation and site-specific selection pressure on the components of all the TCSs in *S. aureus* USA300. Using the NarL family of TCSs and the cocrystal structures of the DesK-DesR from *Bacillus subtilis,* we show the variations and conservation in amino acids mediating the interactions between the DHp and rec domains and how they influence the specificity of phosphotransfer in the TCSs.

## MATERIALS AND METHODS

### Bacterial strains

The *Staphylococcus aureus* strain USA300 JE2 was used as the wild type (WT). The transposon mutants of the TCS components were obtained from the Nebraska Transposon Mutant Library (24) (Table S1). Transposon mutants were chosen based on transposon insertions impacting the gene, specifically those located within the first 60% of the gene length. In-*trans* complemented transposon mutants and plasmids used are detailed in Table S2.

### Complementation of transposon mutants

The primers for cloning the genes for complementation are detailed in Table S3. The open reading frames of the genes were amplified from *S. aureus* USA300 JE2 genomic DNA by PCR, using Q5 Hot Start High-Fidelity DNA Polymerase (New England Biolabs). The resulting PCR products were then ligated into the EcoRI/KpnI sites of the pCM29-sgfp vector using the ClonExpress II One-Step Cloning Kit (Vazyme). These constructs were transformed into *Escherichia coli* DH5α for screening and selection of the correct inserts by PCR. To improve the efficiency of transformation and overcome the restriction barrier, the recombinant plasmids were transformed into and extracted from *E. coli* IM01B. The resulting constructs were subsequently electroporated into the corresponding *S. aureus* transposon mutants, and the transformants were verified by PCR with plasmid-specific primers.

### Human cell lines

The immortalized human keratinocyte cell line HaCaT was cultured in Dulbecco’s modified Eagle’s medium (DMEM) supplemented with 1% penicillin/streptomycin (Sigma) and 10% fetal bovine serum (FBS). The human macrophage cell line THP-1 was cultured in Roswell Park Memorial Institute (RPMI) 1640 media supplemented with 1% penicillin/streptomycin and 10% FBS. Prior to the infection assay, the THP-1 cells were induced to differentiate by adding 10 ng/mL of phorbol myristate acetate for 24 hours. All the cell lines were cultured at 37°C in a 5% CO_2_ incubator.

### Protease assay

The protease assay was performed using the plate diffusion method. Overnight cultures of the *S. aureus* mutants and WT grown in tryptic soy broth (TSB) at 37°C were sub-cultured in fresh TSB to an optical density (OD)_600_ of 0.5. An aliquot of 10 µL of the OD-adjusted cultures was spot inoculated onto 1.5%, wt/vol Mueller-Hinton (MH) supplemented with 2% skim milk (Sigma). The plates were incubated at 25°C for 24 hours. The zone of clearance around the colonies, which is directly proportional to the level of protease activity, was measured.

### HaCaT cell adhesion assay

The HaCaT cell lines were used to test the adhesion ability of the WT and various TCS mutants. The HaCaT cells were seeded in 24-well plates at a density of 1 × 10^6^ cells per mL in DMEM (supplemented with 10%, vol/vol FBS and 1% penicillin/streptomycin). Overnight cultures of *S. aureus* strains in TSB were sub-cultured to an OD_600_ of 1 and diluted in DMEM (supplemented with 10%, vol/vol FBS) to a density of 1 × 10^8^ CFU/mL. The HaCaT cells were infected with bacteria at a multiplicity of infection (MOI) of 10 and incubated for 30 minutes at 37°C and 5% CO_2_. The media were removed, and the cells were washed three times with sterile phosphate-buffered saline (PBS). The cells were lysed with 0.2% triton X-100 (Sigma) in PBS. The lysed cell suspension was serially diluted in PBS, and the number of adherent bacteria was counted by plating and incubating on MH agar for 24 hours at 37°C.

### HaCaT cell viability assay

The test for HaCaT cell viability following the addition of *S. aureus* strains was performed with the tetrazolium dye, MTT (3-[4,5-dimethylthiazol-2-yl]−2,5 diphenyl tetrazolium bromide) assay, which determines mitochondrial activity. The HaCaT cells were seeded at 5,000 cells per well into a 96-well plate. Overnight cultures of *S. aureus* strains grown in TSB at 37°C were sub-cultured into fresh TSB and grown to an OD_600_ of 1 and diluted to 1 × 10^7^ CFU/mL in DMEM. The HaCaT cells were added to *S. aureus* at an MOI of 10 and incubated for 1 hour. Thereafter, the cells were washed with PBS, and tetrazolium dye was added at a final concentration of 5 mg/mL. The plates were incubated for 2 hours at 37°C and 5% CO_2_. The media were aspirated from each well, and the purple formazan crystals were dissolved with dimethyl sulfoxide (DMSO) for 1 hour at 37°C. The absorbance proportional to the viability of the HaCaT cells was measured at 570 nm.

### THP-1 cell infection assay

The differentiated THP-1 cell lines were seeded into 24-well plates at a density of 1 × 10^5^ cells per well in RPMI-1640 (supplemented with 10%, vol/vol FBS, 1% penicillin/streptomycin, and 0.05 mM 2-mercaptoethanol) (Sigma) and incubated for 24 hours in 37°C and 5% CO_2_. Overnight cultures of the *S. aureus* strains in TSB were sub-cultured into fresh TSB and grown to an OD_600_ of 1. The bacteria density was adjusted to 1 × 10^7^ CFU/mL in RPMI-1640 (without penicillin/streptomycin) and added to each well of seeded THP-1 cells at an MOI of 10. The cells were infected for 45 minutes, washed with PBS, and incubated for 30 minutes in RPMI-1640 (supplemented with 100 µg/mL gentamicin and 10% FBS) to kill extracellular bacteria. The cells were washed twice with PBS and lysed with PBS containing 0.2%, vol/vol Triton X-100 in PBS. The lysed cells were serially diluted in PBS, and the number of viable bacteria was counted by plating on MH agar plates for 24 hours at 37°C.

### Nuclease assay

The nuclease assay was performed using the plate diffusion method with the DNase agar (Oxoid). Overnight cultures of the *S. aureus* mutants and WT grown in TSB at 37°C were sub-cultured in fresh TSB to an OD_600_ of 0.5. An aliquot of 10 µL of the OD-adjusted cultures was spot inoculated onto DNase agar and incubated at 37°C for 24 hours. To develop a better contrast between the zone of DNase activity around the colonies, the agar plates were flooded with 1 N Hydrochloric acid and allowed to penetrate the media surface for 5 minutes. The clear zone of polymerized DNA around the colonies indicative of DNase activity was measured.

### Biofilm assay

Overnight cultures of *S. aureus* strains grown in TSB were sub-cultured on fresh TSB and grown to an OD_600_ of 0.5. Briefly, 1 µL of the culture was transferred to 200 µL of TSB in 96-well plates and incubated overnight at 37°C without agitation. The wells were washed to remove the planktonic cells, and the plates were air-dried before staining with 200 µL of 0.1%, wt/vol crystal violet for 10 minutes at room temperature. The wells were washed to remove excess dye and blotted to dry. Each well was filled with 200 µL of DMSO and incubated for 10 min at room temperature to dissolve the dye. The absorbance was measured at 590 nm.

### Hemolysis

The hemolysis assay was performed using the plate diffusion method. A volume of 10 µL of *S. aureus* strains grown in TSB to an OD_600_ of 0.5 was spot inoculated onto blood agar plates and incubated at 37°C for 24 hours and an additional 4°C incubation for 24 hours. The diameter of the hemolytic zone was measured after 48 hours.

### Protein A assay

Costar 96-well enzyme-linked immunosorbent assay (ELISA) plates were coated with 10 µg of anti-protein A antibodies (ACRIS) in PBS overnight at 4°C, washed three times with PBS-T (100 µL PBS containing 0.05%, vol/vol Tween) and air-dried. Additionally, the plates were blocked with 1% bovine serum albumin (BSA) for 2 hours at 37°C.

Overnight cultures of *S. aureus* strains grown in TSB at 37°C and 200 rpm were sub-cultured into fresh TSB and grown to an OD_600_ of 1.0. Briefly, 100 µL of the bacteria was added to the wells and incubated at 37°C for 1 hour. The plates were washed twice with 200 µL of PBS-T, fixed with 100 µL of 4% paraformaldehyde for 20 minutes at room temperature, washed twice with ddH_2_O, and air-dried.

The adhered bacterial cells in the wells were stained with 150 µL of 0.1% crystal violet and incubated at room temperature for 5 minutes. The plates were then washed twice with ddH_2_O, and the crystal violet was solubilized with 200 µL of 30% acetic acid at room temperature for 15 minutes. The absorbance was measured at OD_595_. The protein A null mutant Δ*spa* was used as a negative control (Fig. S1).

### Bacterial binding to immobilized fibronectin and fibrinogen

Costar 96-well ELISA plates were coated with 10 µg of fibronectin (Sigma) for the fibronectin-binding assay and 10 µg of fibrinogen (Sigma) for the fibrinogen-binding assay in PBS overnight at 4°C. The wells were washed three times with PBS-T and air-dried for 1 hour. The plates were blocked with 1% BSA for 2 hours at 37°C.

Overnight cultures of *S. aureus* strains grown in TSB at 37°C and 200 rpm were sub-cultured into fresh TSB and grown to an OD_600_ of 1.0. Briefly, an aliquot of bacteria was incubated at 37°C in 10% FBS in TSB for 30 minutes. Following treatment, 100 µL of the bacteria suspension was added to the fibronectin- or fibrinogen-coated plate and incubated at 37°C for 1 hour.

The plates were washed with 200 µL of PBS-T and fixed with 100 µL of 4% paraformaldehyde for 20 minutes at room temperature. The plates were washed twice with ddH_2_O after fixing and air-dried.

To visualize the attached bacterial cells, 150 µL of 0.1% crystal violet was added to each well and incubated at room temperature for 5 minutes. The plates were washed twice with ddH_2_O and air-dried. To solubilize the crystal violet, 200 µL of 30% acetic acid was added to each well and incubated at room temperature for 15 minutes. The absorbance was measured at OD_595_. The fibronectin-binding protein AB mutant Δ*fnbpAB* was used as a negative control (Fig. S1).

### Identification of two-component systems

The software HMMER (25) was used with the *E*-value cutoff of 0.01 to identify the two-component systems present in the whole genome of the *S. aureus* USA300_FPR3757. The model for HMMER was created using sequences of TCSs published in the KEGG pathway database (https://www.genome.jp/brite/ko02022).

### Analysis of selection pressure in TCS genes

The analysis of site-specific selection pressure was analyzed using the method described in reference (26) with minor modifications. The *S. aureus* genomes were downloaded from the NCBI database. The Refseq, complete, and annotated genomes were filtered based on the deposited genomes between 2000 and 2023. The compiled database was composed of the coding sequences (CDS) and the corresponding amino acid sequences in FASTA format. Orthologs of the TCS in *S. aureus* genomes were determined using the Orthofinder software (27) with the *S. aureus* USA300 TCSs as reference sequences. For each TCS HK and RR, duplicate sequences were removed using the SeqKit program (28), and the orthologs were aligned by MUSCLE (29). The codon alignment was generated using pal2nal (30), and the aligned sequences were trimmed with trimAI (parameter:gappyout) (31). The treefile was generated from the aligned sequence using iqtree (parameter: st = DNA m = GTR + G4 nt = 1 fast) (32). The mean posterior probabilities for selection pressure per site across each gene in the TCS were calculated by Fast, Unconstrained Bayesian AppRoximation for inferring selection (FUBAR) (33).

### Protein modeling and phylogenetic analysis

The modeling of the *S. aureus* NarL HKs and RRs was generated with SWISS-MODEL (34). To determine the interacting residues between each NarL TCS cognate RR and HK, the models were superimposed onto the DesK-DesR cocrystallized structure (PDB: 5IUJ) (35) using PyMOL (36). Amino sequence alignments of the TCS RR and HK domains between different *Staphylococcus* strains and species, as well as *B. subtilis,* were performed with MUSCLE (29), and phylogenetic trees were constructed with IQ-TREE (32).

### Co-evolution analysis of TCS in *S. aureus* USA300

The analysis of coevolving residues between the DHp and the Rec domains was performed following computational methods described in reference (37) with minor modifications. The cognate pairs for all TCSs in the 1,000 *S*. *aureus* genomes downloaded from the NCBI database were selected for the Orthofinder analysis using the cognate TCS pairs from the *S. aureus* USA300 as references. Duplicate TCS pairs were removed using the SeqKit program (28). For the HKs, the transmembrane and sensor domains were removed, and for the RRs, the output domains were removed. The remaining DHp and Rec domains were concatenated into a single sequence and aligned using MUSCLE (29). Columns in the alignment containing more than 10% gaps were eliminated from consideration. The mutual information analysis was performed using the online software MISTIC (38). The coevolving pairs were chosen based on an adjusted score of 3.5 or higher, ranking among the top 5–50 coevolving residues and those with the highest selection pressures. Although additional clusters of coevolving residues can be found at lower thresholds, this analysis focuses on the top 90% of coevolving residues within the cognate pairs (29, 32, 34–36).

### Statistical analysis

Normality within the data sets was assessed using the Shapiro-Wilk test, where *P*-value < 0.05 indicates that the data do not follow a normal distribution. Subsequently, Levene’s test for equal variance was conducted to determine the appropriate test for comparing groups, either the Mann-Whitney *U* test for non-normally distributed data or Welch’s corrected *t*-test for normally distributed data with unequal variances.

## RESULTS

### Phylogenetic and domain analysis of TCSs in *Staphylococcus aureus*

To identify the TCSs in *S. aureus* USA300, the genome was scanned using an HMMER model developed from the domains associated with TCSs from both Gram-positive and negative bacteria. This revealed 16 TCSs belonging to NarL, OmpR, AraC, LytTR, and an unclassified member of TCS. Orthologs of the TCS were also identified in the *S. aureus* reference strains NCTC8325 and MSSA476 (Table 1).

**TABLE 1:** Two-component systems identified in *S. aureus* USA300 and their orthologs in *S. aureus* reference strains NCTC8325 and MSSA476

Family	TCS	USA300_FPR3757	NCTC8325	MSSA476
		(SAUSA300_)	(SAOUHSC_)	
AraC	HptSR	0217/0218	00184/00185	SAS0198/SAS0199
NarL	VraSR	1865/1866	02097/02098	SAS1806/SAS1807
NarL	NreBC	2337/2338	02675/02676	SAS2282/SAS2283
NarL	DesKR	1219/1220	01313/01314	SAS1261/SAS1262
NarL	Hypothetical	1798/1799	01980/01981	SAS1769/SAS1770
OmpR	SrrAB	1441/1442	01585/01586	SAS1431/SAS1432
OmpR	SaeSR	0690/0691	00714/00715	SAS0670/SAS0671
OmpR	ArlSR	1307/1308	01419/01420	SAS1357/SAS1358
OmpR	HssSR	2308/2309	02643/02644	SAS2252/SAS2253
OmpR	GraSR	0645/0646	00665/00666	SAS0624/SAS0625
OmpR	BraSR(NsaSR)	2558/2559	02955/02956	SAS2510/SAS2511
OmpR	VicKR(WalKR)	0020/0021	00020/00021	SAS0018/SAS0019
OmpR	PhoPR	1638/1639	01799/01800	SAS1620/SAS1621
OmpR	KdpDE	2035/2036	02314/02315	SAS1983/SAS1984
LytTR	AgrAC	1991/1992	02264/02265	SAS1943/SAS1944
LytTR	LytST	0254/02545	00230/00231	SAS0237/SAS0238
Unclassified	YycHI	0022/0023	00022/00023	SAS0020/SAS0021

**Fig 1:**
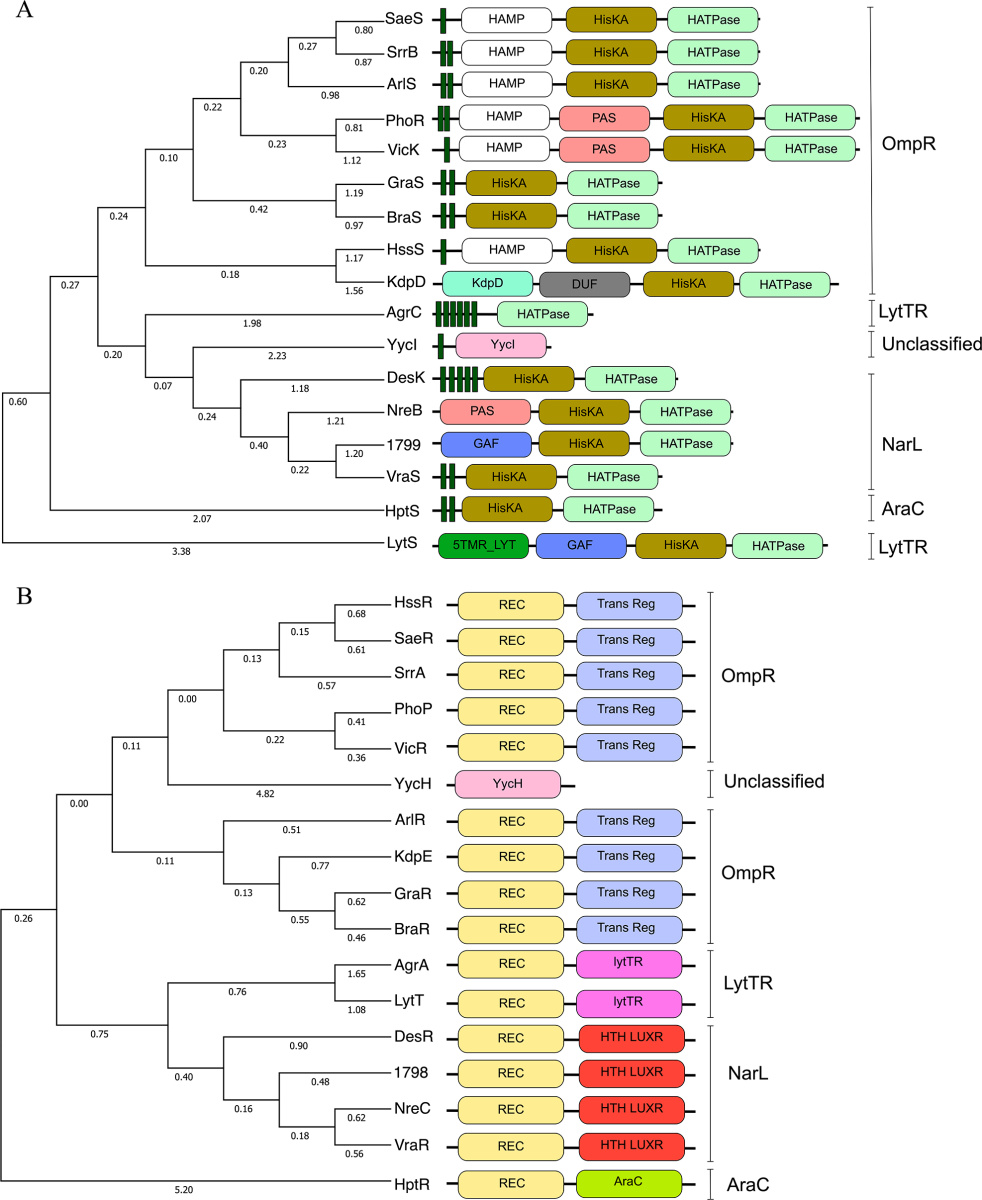
Phylogenetic and domain analysis of TCSs in *Staphylococcus aureus*** (A**) histidine kinases and (**B**) response regulators. Phylogenetic analysis was performed using the amino acid sequences of the components from the USA300-FPR3757 genome, employing maximum likelihood testing and a bootstrap value of 1,000. Domains corresponding to the TCS components are indicated by labeled, colored rectangular blocks, while vertically labeled lines show families. The TMR domains of the HKs are shown as narrow green rectangular blocks.

Putative domain search using SMART (39) showed the presence of domains such as the transmembrane (TMR), HisKA (dimerization and phosphoacceptor), and the HATPase (histidine kinase-like ATPase) domains in most of the HKs. Some of the HKs contained additional domains such as the HAMP, PAS, GAF, and DUF4118 domains commonly found between the transmembrane and the HisKA domains in HKs (Fig. 1A). The unclassified HK YycI possessed a transmembrane domain and a YycI domain, whereas the AgrC possessed six TMR and a HATPase domain. The NreB and the SAUSA300_1799 lacked the TMR, whereas the four predicted TMR in the KdpD overlap with the DUF domain. All the RRs contained the common Rec (phosphoacceptor) domain in all the RRs with either of the effector binding domains such as the *Trans* Reg, LytTR, HTH-LUXR, and AraC in different RRs. These domains were absent in YycH (Fig. 1B). YycH and YycI are accessory components to the essential TCS, WalKR. While they are not classified within the traditional TCS framework, their inclusion in this analysis was based on their direct role in stimulating and fully activating the WalKR system (40).

Phylogenetic analysis based on the amino acid sequences of the TCSs demonstrated high relatedness between the HKs and RRs within their respective families. This clustering was more evident in the RRs, as they share a more conserved domain architecture. Interestingly, even though some HKs possessed additional domains, they still exhibited close relatedness within their families, except for the LytTR and unclassified HKs (Fig. 1). This observation suggests that, despite the presence of extra domains, the core structural features of these TCSs are conserved across their respective families.

### Analysis of virulence determinants in transposon mutants of the TCSs in *S. aureus*

The common domain architecture and relatedness between individual HKs and RRs within TCSs can influence their regulation of virulence determinants and lead to possible cross-regulation of biological pathways. To investigate how the conservation and clustering of HKs and RRs influence their control of virulence determinants, the transposon mutants of the TCS components were examined for their effect on biofilm formation, hemolytic activity, protease activity, protein A production, nuclease production, fibronectin and fibrinogen binding, and their ability to infect, adhere, and kill human cell lines.

**Fig 2:**
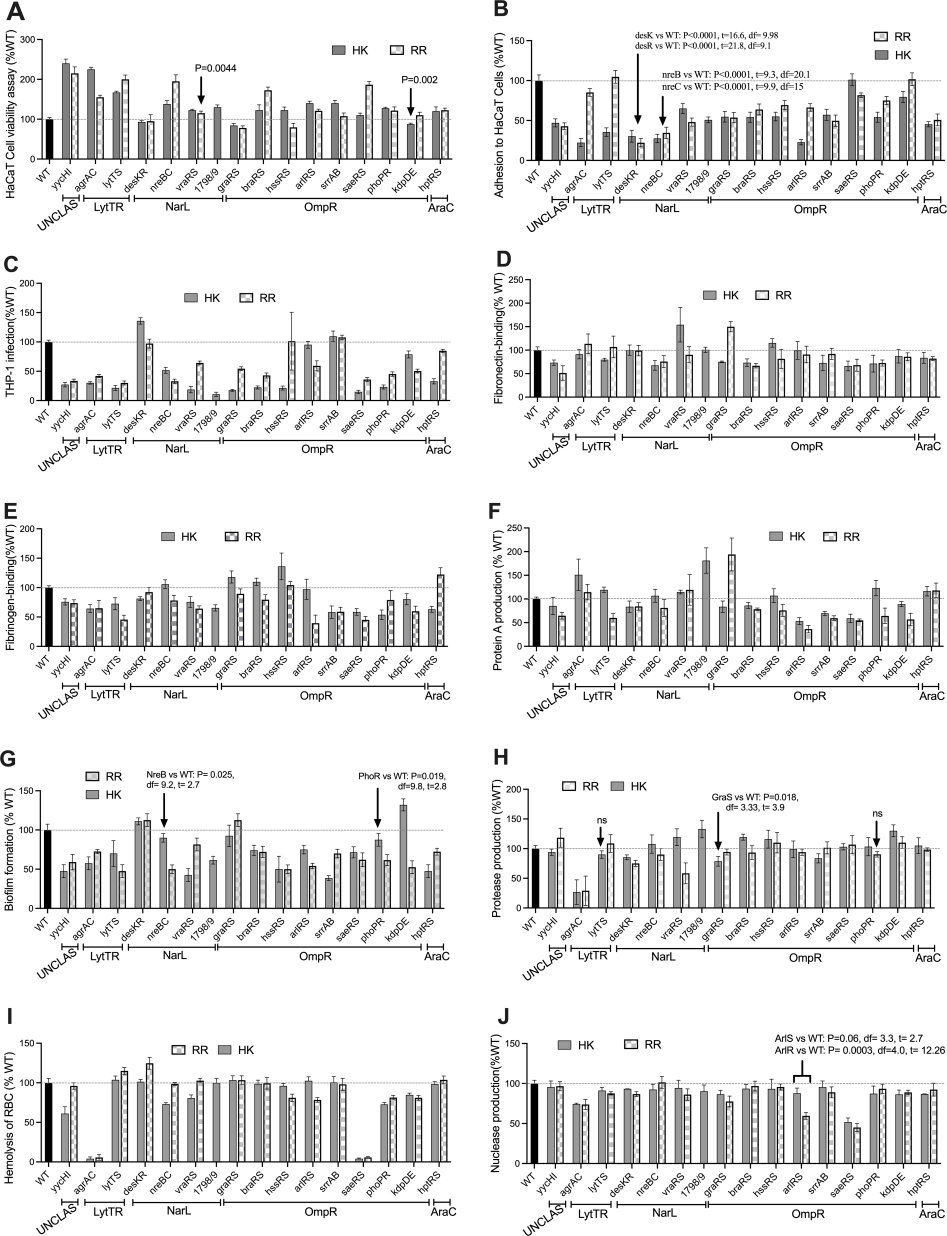
Analysis of virulence determinants in transposon mutants of the TCSs in *S. aureus*. Virulence profiles of the HKs and RR mutants grouped based on the family. (**A**) HaCaT cell viability assay, (**B**) adhesion of bacteria to HaCaT cells, (**C**) bacterial infection of THP-1 cells, (**D**) fibronectin binding, (**E**) fibrinogen binding, (**F**) protein A production, (**G**) biofilm formation, (**H**) protease production, (I) hemolysis of red blood cells, and (J) nuclease production of the *S. aureus* TCS component transposon mutants. The cognate pairs of HKs and RRs are grouped based on their respective family. The production of the virulence phenotypes is expressed as a percentage of the wildtype normalized to 100%. All data represent mean ± SD of *n* = 4 independent experiments. *P*-value < 0.05 is considered significant.

The TCS mutants YycIH, AgrAC, LytTS, NreBC, BraRS, ArlRS, PhoPR, HptRS, and VraRS were significantly less cytotoxic for HaCaT cell lines than the WT (Fig. 2A). In contrast, the GraSR, HssR, and KdpE mutants displayed increased cytotoxicity, while the remaining TCS mutants showed either marginal reductions or no significant differences in cytotoxicity when one of the components was inactivated. In some of the TCS, mutations in either the HK or the RR resulted in varying effects on their cytotoxicity. For instance, in SaeRS, the mutation of the RR significantly reduced cytotoxicity, while gene inactivation of its cognate HK had a similar effect as the WT. These observations may suggest the possibility of crosstalk activities among different HKs or that the RR SaeR can also respond to other regulatory systems (Fig. 2A).

Moreover, the adhesion of *S. aureus* to HaCaT cells was generally diminished among the TCS mutants, except for HK SaeS and the RRs LytT and KdpE (Fig. 2B). The DesKR and NreBC showed the most significant reduction in adhesion to HaCaT cells. Additionally, compared to the WT, the TCS mutants mostly exhibited reduced infection in THP-1 cell lines except for the DesKR, SrrAB, HssR, and ArlS (Fig. 2C).

*S. aureus* cell wall-anchored factors facilitate bacterial attachment to host tissue, promoting colonization and evasion of host immune factors (2, 3). While previous reports have indicated that SaeRS regulates fibronectin and fibrinogen binding (41, 42), we observed reduced binding to immobilized fibronectin in the mutants of TCSs YycHI, NreBC, BraSR, and SaeRS (Fig. 2D). In contrast, mutants of AgrAC, DesKR, VraSR, HssSR, and ArlSR showed no difference or increased binding to fibronectin compared to the WT. Distinct variations in binding to fibronectin were observed among some mutant RRs and HKs, including LytS, HptR, and PhoR (PhoR vs WT: *P* = 0.09, df = 2.6, and *t* = 2.14) when compared to their corresponding cognate pairs (Fig. 2D). A particularly significant deviation was noted within the GraSR system’s HK-RR pair. The GraR mutant (GraR vs WT: *P* = 0.004, df = 3.5, and *t* = −6.5) demonstrated increased binding to fibronectin compared to the WT, whereas the GraS mutant (GraS vs WT: *P* = 0.01, df = 2.2, and *t* = 6.12) revealed a diminished binding compared to the WT.

From our assay, only YycHI and SaeRS influenced adhesions to both immobilized fibrinogen and fibronectin (Fig. 2D and E). Additionally, we observed decreased binding to fibrinogen in the SrrAB, AgrAC, LytTS, VraRS, and KdpDE compared to the WT (Fig. 2E). The AgrAC, typically a negative regulator of fibronectin binding (43) (Fig. 2D), exhibited a positive regulatory effect on fibrinogen binding. Interestingly, both HK-RR transposon mutants of HssRS showed a marginal increase (HssS vs WT: *P* = 0.01, df = 5.1, and *t* = −3.8) or WT-level binding (HssR vs WT: *P* = 0.19, df = 7.48, and *t* = −1.2) to fibrinogen, while individual RRs HptR and DesR, and HKs BraS, NreB, and ArlS exhibited increased or similar binding profiles to fibrinogen compared to the WT (Fig. 2E). Although HssRS is known for its role in sensing heme concentration, but there is no direct experimental evidence of its influence on fibrinogen and fibronectin binding. HssRS controls *htr*A expression, which counteracts heme toxicity. Mutations in *hssR* and *htrA* lead to *S. aureus* hypervirulence via heme buildup, while *htrA* mutants upregulate fibronectin and fibrinogen-binding proteins (44–46), which could explain the increased binding of HssRS mutants to fibrinogen and fibronectin (Fig. 2D and E).

In addition to SaeRS and ArlRS, which positively regulate binding to bacterial surface-associated Protein A (47), mutations of SrrAB showed a significant decrease in binding to immobilized anti-Protein A antibodies compared to the WT (Fig. 2F). This is possibly due to the SrrAB negative regulation of the *agr* locus (48). In agreement, the mutation of the HK-RR of AgrAC showed increased binding to the immobilized anti-protein A antibodies (47) (Fig. 2F). The mutants of the HK-RR pairs of KdpDE (KdpD vs WT: *P* = 0.05, df = 3.7, and *t* = 2.8) and the BraSR (BraS vs WT: *P* = 0.04, df = 3.32, and *t* = 3.12) showed a marginal difference in binding to anti-protein A antibodies. VraRS, HptRS, and the individual RR GraR and HKs LytS, SAUSA300_1799, and PhoR displayed increased binding to anti-protein A antibodies compared to the WT (Fig. 2F).

Biofilm formation in *S. aureus* is a complex and dynamic process regulated by a network of interconnected TCSs. Mutations of the TCSs, except for DesKR, GraSR, and the HK KdpD were found to reduce biofilm formation (Fig. 2G). TCSs like ArlRS, AgrAC, SrrAB, SaeRS, and LytTS play critical roles in modulating the expression of surface proteins, extracellular matrix components, and quorum-sensing molecules, which contribute to biofilm initiation, maturation, and dispersal (49).

While the influence of GraS on protease production is most notable under acidic pH conditions (50), we found a 15%–20% reduction in protease activity for the *graS* mutant compared to the WT in cation-adjusted MH at pH 7. Interestingly, there was no significant difference in protease production between the *graR* mutant and the WT (Fig. 2H). Although ArlRS and SaeRS have been reported to control various genes in protease loci (51), our analysis showed no impact on protease production for their corresponding mutants. In contrast, TCS AgrAC mutants demonstrated the most substantial effect on protease production. Furthermore, mutations in both DesKR components led to a 15%–20% decrease in protease production compared to the WT level. Additionally, while the VraR mutant exhibited a significant reduction in protease production, its sensor kinase VraS showed no discernible difference from the WT (Fig. 2H).

Hemolysis was most notably reduced in the AgrAC and SaeRS mutants. Additionally, the PhoPR and KdpDE mutants displayed decreased hemolysis compared to the WT (Fig. 2I). Furthermore, mutants of the HKs YycI, NreB, VraS, and the RRs HssR and ArlR showed reduced hemolysis compared to their cognate pairs, which were similar to the WT levels (Fig. 2I). Both SaeRS and AgrAC TCSs are known to influence hemolysis in *S. aureus* (42). Although ArlSR has been shown to indirectly affect hemolysis by regulating SaeRS via ScrA (52), our study only observed reduced hemolysis in the ArlR mutant (ArlR vs WT: *P* < 0.0001, df = 7.6, and *t* = 8.6) (Fig. 2I).

Nucleases play a critical role in evading the host immune system by degrading host DNA, as well as in nutrient acquisition and biofilm maintenance (53). Among the TCS mutants, SaeRS exhibited the most significant decrease in nuclease production, followed by ArlR, AgrC, and, to a lesser extent, the GraRS system (Fig. 2J). This is in agreement with previous studies that demonstrated the influence of SaeRS and ArlRS on nuclease production (54, 55).

Considering the regulatory impacts of the TCS components on virulence, we explored TCS pairs that exhibit similar cross-regulation of virulence profiles. Principal component analysis (PCA) of the virulence profiles in the HK and RR mutants identified five clusters that displayed limited similarity in their family classifications. Cognate pairs that showed similarities in virulence regulation included the SrrAB, SaeRS, YycHI, BraSR, PhoPR, NreBC, AgrAC, and DesKR (Fig. S2A and B). Among these gene sets, the SaeRS and AgrAC have global regulons and influence a subset of genes controlled by other TCSs in *S. aureus* (56). Furthermore, the AgrAC formed a separate cluster, suggesting its ability to influence the production of one or more virulence determinants independently or significantly. Despite belonging to the same family as LytTR (Fig. 1), the AgrAC did not cluster with LytT or LytS (Fig. S2). HKs, including KdpD, ArlS, and DesK, and the RRs, HptR, HssR, and DesR, demonstrated similar virulence regulatory profiles as the WT. These results suggest that possible cross-regulation between the TCS in regulating biological pathways is crucial for the bacteria infection potential. Also, the observed variations in virulence regulatory profiles among TCS components and the clustering noted from the PCA show significant crosstalk among different TCSs. Such interactions amplify or mitigate the bacterium’s virulence, shaping the overall pathogenic potential of *S. aureus*.

The study identified virulence profiles using transposon mutants targeting the TCS HKs and RRs from *S. aureus*. To confirm the validity of these virulence profiles and address contrasts in virulence determinant regulation between cognate TCS pairs, we focused on three TCSs: ArlRS, SaeRR, and SrrAB. These were chosen for their high sequence homology and phylogenetic relationship within the OmpR family (Fig. 1) and their varied regulatory effects of the cognate HKs and RRs on specific virulence determinants, such as hemolysis of RBC, protein A, and nuclease production (Fig. 2). The HKs and RRs of the SaeRS and SrrAB displayed similar regulatory patterns for virulence determinants (Fig. 2; Fig. S2), whereas the HK and RR of the ArlSR TCS exhibited divergent effects on regulating some virulence factors (Fig. 2; Fig. S2), suggesting potential cross-regulation with other TCS and regulatory genes (22, 57, 58). We employed in-*trans* complementation for the ArlRS, SrrAB, and SaeRS components to substantiate these findings within their mutant backgrounds. Regarding protein A production, its defective levels in the transposon mutants of all three TCS components, SaeRS, ArlRS, and SrrAB, were rescued to the WT levels in their respective complemented strains (Fig. S1D).

Furthermore, the transposon mutants of the SaeR, SaeS, and ArlR, which were both nuclease and hemolysis defective, were rescued to the WT levels when in-*trans* complemented with their corresponding genes (Fig. S1E and F). Under the conditions tested, the SaeRS, ArlSR, and SrrAB TCS pairs displayed analogous regulatory functions over virulence determinants, such as protein A, indicating a possibly conserved regulatory role (Fig. 2; Fig. S1D) (59). In contrast, the ArlSR TCS shows varying effects on other virulence factors, suggesting the possibility of nonspecific interactions or crosstalk with other regulatory mechanisms. Restoring protein A levels in HK and RR mutants of all three TCSs to the WT level in the complemented strains shows that the respective mutations directly impact virulence determinants.

### Evolution and selection of TCS in *S. aureus* strains

The role of TCS in virulence regulation and their conservation in bacterial genomes make them potential targets for antimicrobial agents. Investigating selection pressure across TCS gene sites provides insights into the evolutionary constraints and functional diversity of the TCS. To further explore the selection pressure, evolutionary stability, and conservation of TCSs in *S. aureus* strains, we conducted an evolutionary test of selection pressure using phylogenies of TCSs derived from 1,000 complete and annotated *S. aureus* genomes. For this analysis, we used FUBAR, which applies the Bayesian approach to infer nonsynonymous (dN) and synonymous (dS) substitution rates on a per-site basis for a given coding alignment and corresponding phylogeny (33). Orthologs of each TCS RR and HK from the 1,000 *S*. *aureus* genomes (from 2000 to 2022 available at NCBI) were used for codon alignment and phylogeny. More than 86% of the analyzed genomes contained all TCSs present in USA300_FPR3757, indicating high evolutionary conservation (Fig. S3).

**Fig 3:**
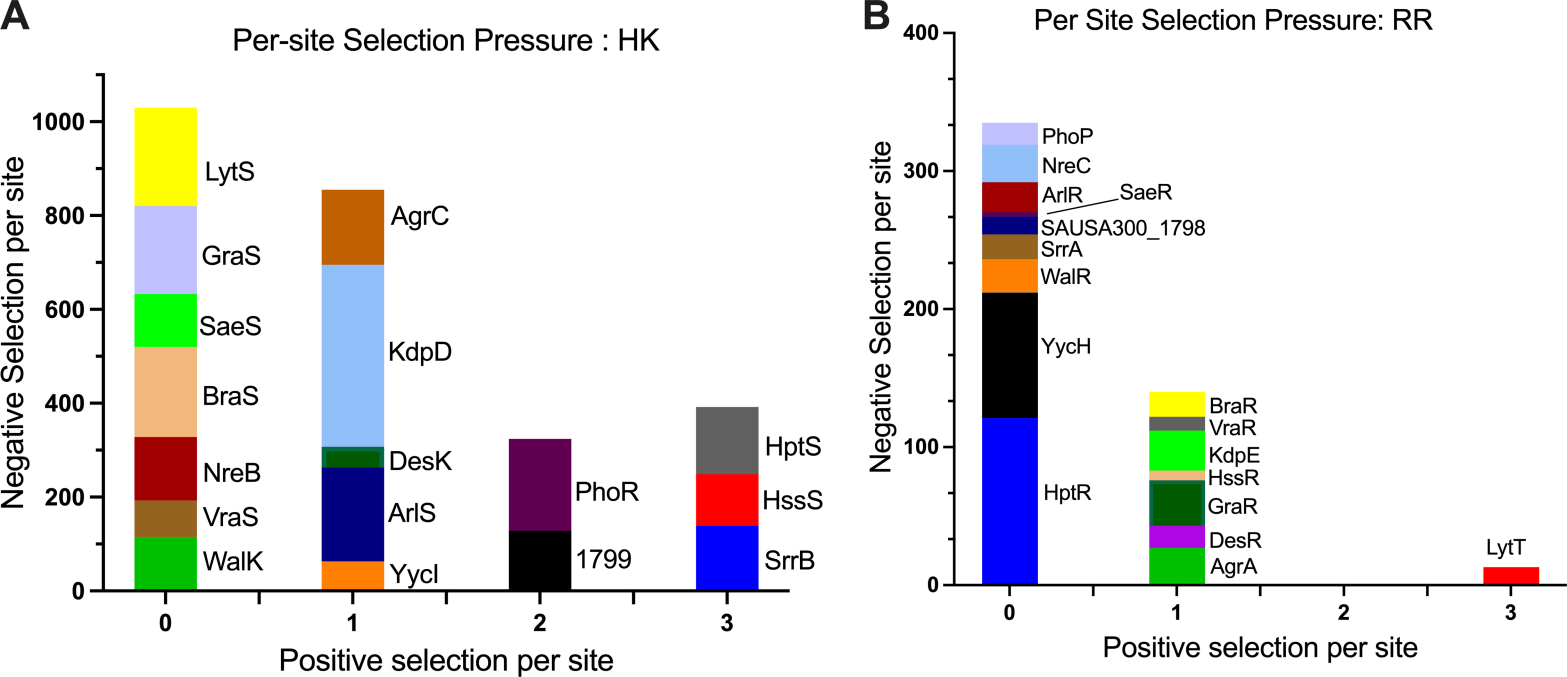
The selection pressure per site in the HKs and RRs of the TCS in *S. aureus*. The number of sites under negative selection pressure (*y*-axis) and positive selection pressure (*x*-axis) in the (**A**) HKs and (**B**) RRs of the TCS from the *S. aureus* genomes.

Overall, we noted more pronounced negative (purifying) selection pressures on the HKs in comparison to the RRs (Table S4). No positive (diversifying) selection was identified for residues in HKs WalK, SaeS, VraS, NreB, GraS, BraS, and LytS. One site under positive selection pressure was identified in the YycI, ArlS, DesK, KdpD, and AgrC, while SAUSA300_1799, PhoR, SrrB, HssS, and HptS had two or three sites undergoing positive selection pressure (Fig. 3A; Table S4).

Fewer sites under positive and negative selection pressures were observed in the RRs compared to their cognate HKs. Most RRs, including PhoP, HptR, WalR, YycH, ArlR, SrrA, SAUSA300_1798, SaeR, and NreC, had no residues under positive selection pressure (Table S4). In contrast, RRs GraR, HssR, KdpE, VraR, BraR, AgrA, and DesR had one site with inferred positive selection, while LytT had three sites with positive selection pressure (Fig. 3B; Table S4). In both the HKs and RRs, no sites under positive selection were observed in the TCSs WalKR, NreBC, and SaeRS. This finding aligns with the expectations for WalKR, which is an essential TCS (60). Although the NreBC and SaeRS TCSs are not considered essential TCSs and their cognate pairs are not subject to positive or negative evolutionary selection pressures (Table S4), they demonstrate high specificity in regulating virulence factors (Fig. 2; Fig. S2).

### Coevolution of specificity determinants in NarL TCS

Given the selection pressures across the sites of HKs and RRs, we aimed to further elucidate the evolutionary relationship between the interacting residues of cognate TCS pairs. Given the importance of HK-DHp and RR-Rec domain residue interactions in TCS specificity (37), we investigated the evolutionary link between these interacting residues in TCS components, exploring the potential for TCS crosstalk.

**Fig 4:**
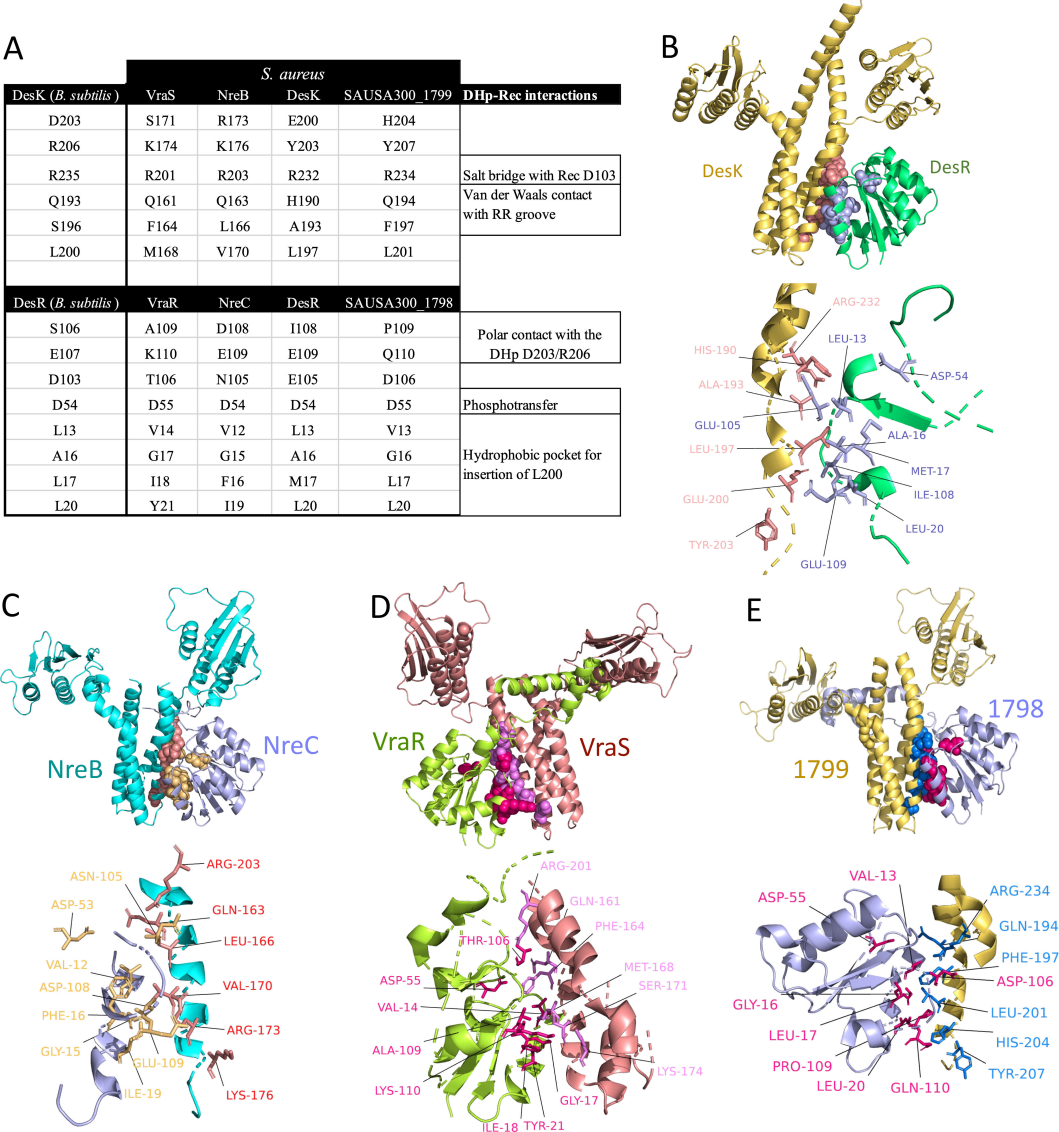
(A) Interacting residues from *B. subtilis* DesK and DesR, as well as *S. aureus* HKs and RRs from the NarL family, are displayed. The selected residues from *S. aureus* are based on their alignment with the interacting residues of DesK (DHp) and DesR (Rec) domains. Each residue is shown with its amino acid and position within the specific protein. (B) Superimposing the RR and HK domains from the modeled *S. aureus* DesK and DesR onto the cocrystal structure of the *B. subtilis* DesKR demonstrated high similarities in alignment (1.993 Å RMSD aligning 2,296 atoms of the HK and 0.351 Å RMSD aligning 626 atoms of the RR). (C) The NreB-NreC model using the *B. subtilis* DesKR revealed very similar structures, with 2.114 Å RMSD aligning 1,959 atoms of the HK and 0.402 Å RMSD aligning 601 atoms of the RR. (D) Superimposition of the modeled VraRS with the *B. subtilis* DesK-DesR complex showed high similarity in alignment, with 2.487 Å RMSD aligning 2,006 atoms of the HK and 0.322 Å RMSD aligning 577 atoms of the RR. (E) Superimposition of the modeled SAUSA300_1798/9 with the *B. subtilis* DesK-DesR cocrystal structure showed high similarity in alignment, with 2.114 Å RMSD aligning 1,959 atoms of the HK and 0.402 Å RMSD aligning 601 atoms of the RR.

We employed the NarL TCS DesKR cocrystal structure from *Bacillus subtilis*_168 (35) as a modeling proxy to illustrate the variations in specificity-determining residues between the DHp and Rec of the NarL TCS in *S. aureus* consisting of DesKR, VraRS, NreBC, and SAUSA300_1798/1799. Among these members, NreBC, VraS, and SAUSA300_1798 exhibit no sites under diversifying selection (Fig. 3; Table S4), which suggests strong evolutionary stability and indicates functional constraint. Additionally, cognate pairs of the DesKR and the NreBC showed similarities in the control of virulence determinants (Fig. 2; Fig. S2), which signifies the conservation of signal transduction within the TCSs. Contrastingly, the HK VraS and its associated RR VraR displayed divergent virulence regulatory profiles, suggesting potential crosstalk with other systems (Fig. 2; Fig. S2). While VraS and SAUSA300_1799 exhibit similar virulence profiles, the lack of a transposon mutant for RR SAUSA300_1799 hinders a comprehensive comparison for potential cross-regulation in virulence between the two systems. Through amino acid sequence alignment between *B. subtilis* DesKR and *S. aureus* NarL TCSs, we mapped residues in the DHp (six residues) and Rec (eight residues) domains that mediate interactions. The interacting residues varied between the various *S. aureus* NarL TCSs and the DesKR from *B. subtilis* (35) (Fig. 4A; Fig. S4 and S5). Superimposition of the *S. aureus* NarL TCSs demonstrated a similar localization of interacting residues within the DHp and Rec domains, consistent with the arrangement observed in the *B. subtilis* DesKR cocrystal structure (Fig. 4B through E; Fig. S6). We did not analyze polar interactions between the residues, as these models are only derived from alignment with predetermined structures and may not provide accurate distances.

**Fig 5:**
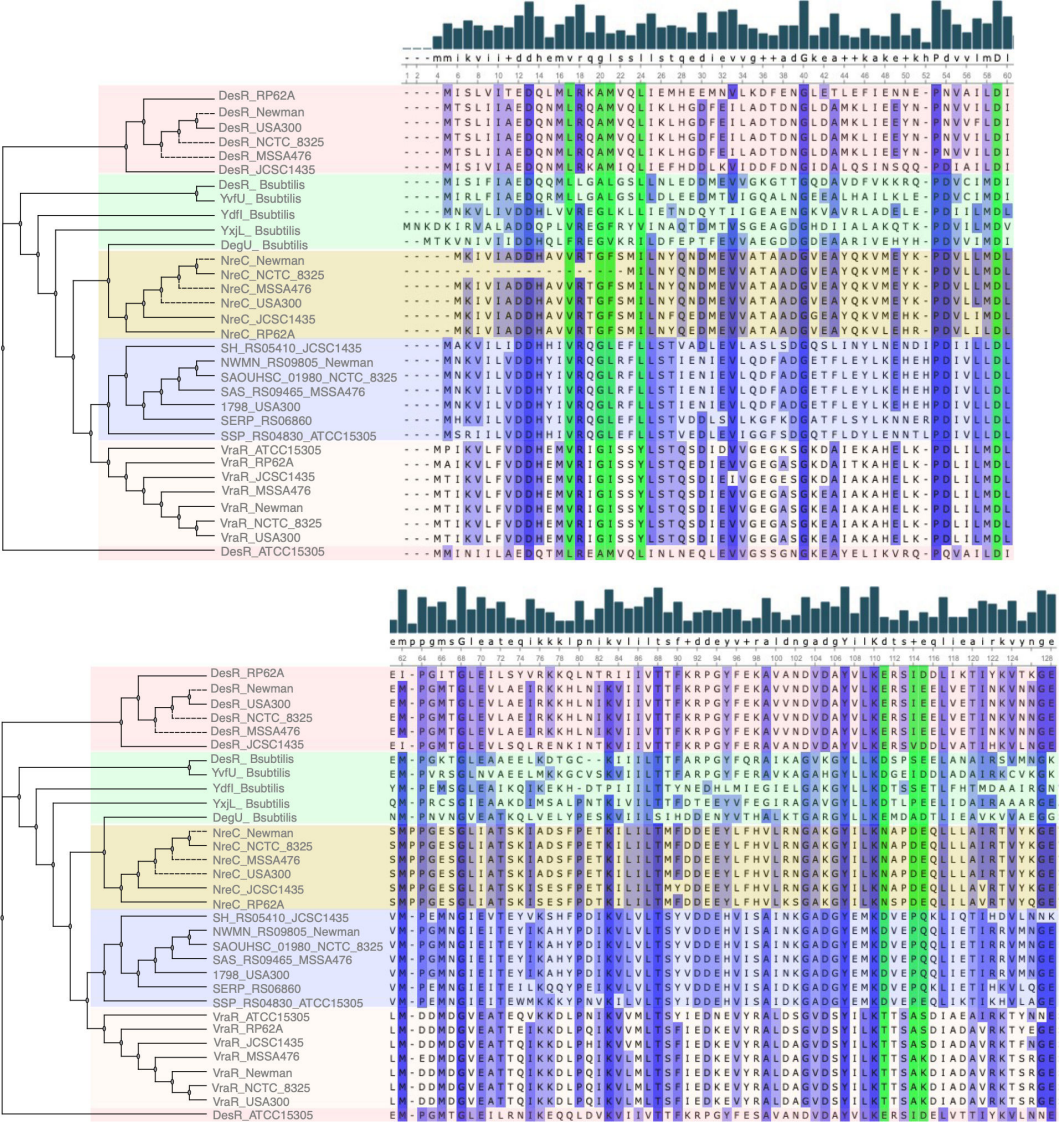
The phylogenetic analysis and sequence alignment illustrate the relationships between the NarL TCS RR Rec domains from *Staphylococcus* spp. and *B. subtilis*. Gaps introduced in the sequence alignment serve to maximize alignment and are represented as dashes. The green vertical shading indicates the aligned residues that interact with the DHP domain. The blue vertical shading, with increasing intensity, signifies conserved regions. The degree of consensus sequence is depicted by the bar chart, with uppercase residues representing highly conserved areas and lowercase residues for less conserved regions. The horizontal-colored shading demonstrates the clustering based on the percent identity matrix of the aligned amino acid sequences of the full-length RR from each TCS (Fig. S4B). Groups under the same horizontal colored shading belong to the same cluster. Phylogenetic analysis was performed with MUSCLE with a bootstrap value of 1,000.

**Fig 6:**
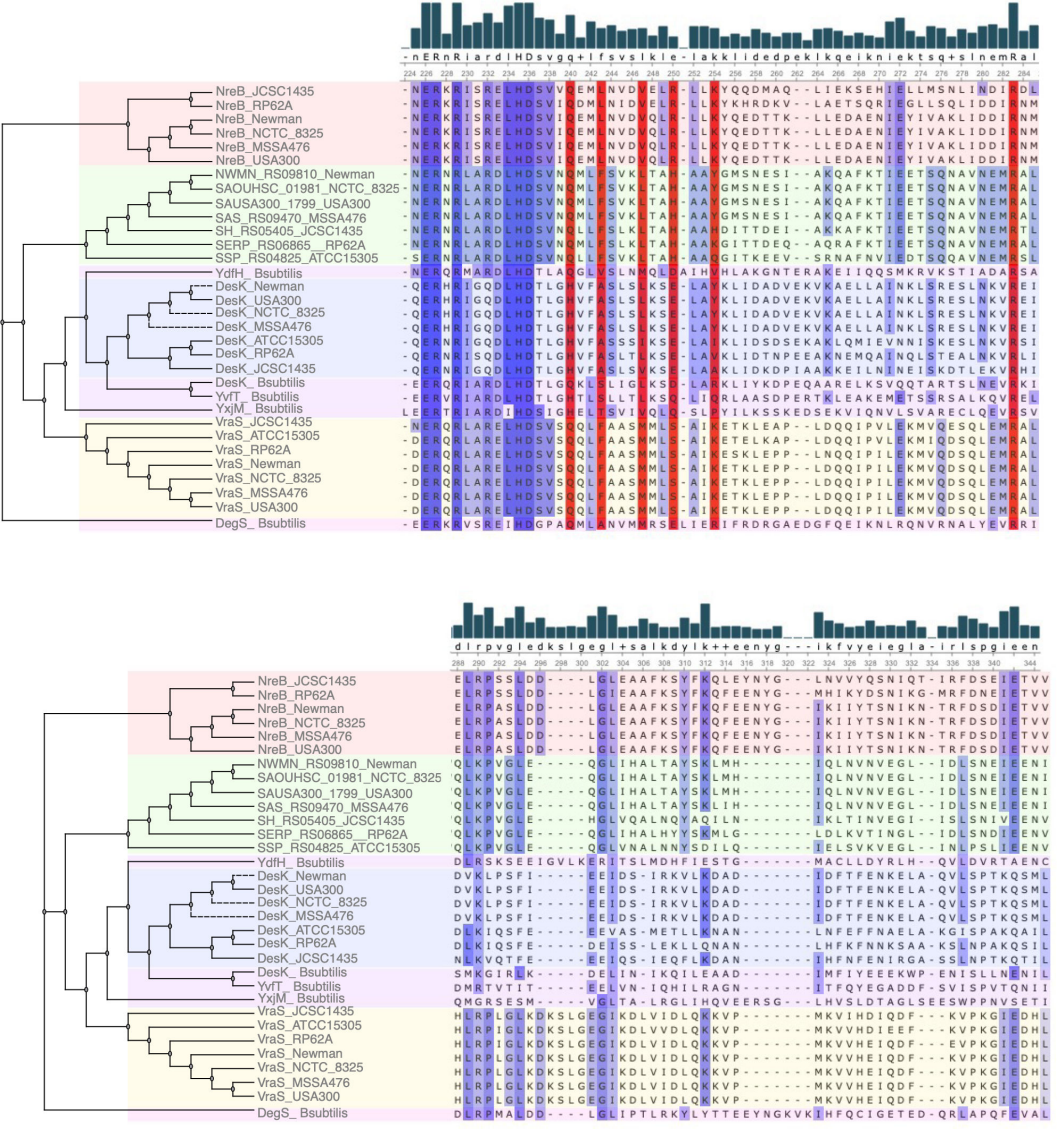
The phylogenetic analysis and sequence alignment illustrate the relationships between the NarL TCS DHp domains from *Staphylococcus* spp. and *B. subtilis*. Gaps introduced in the sequence alignment serve to maximize alignment and are represented as dashes. The red vertical shading indicates the aligned residues that interact with the Rec domain. The blue vertical shading, with increasing intensity, signifies conserved regions. The degree of consensus sequence is depicted by the bar chart, with uppercase residues representing highly conserved areas and lowercase residues for less conserved regions. The horizontal-colored shading demonstrates the clustering based on the percent identity matrix of the aligned amino acid sequences of the full-length HK from each TCS (Fig. S4A). Groups under the same horizontal-colored shading belong to the same cluster. Phylogenetic analysis was performed with MUSCLE with a bootstrap value of 1,000.

Investigation of the conservation of interacting residues among the NarL TCSs in *Staphylococcus* species (*S. aureus* Newman, *S. aureus* USA300, *S. aureus* MSSA476, *Staphylococcus epidermidis*, *S. aureus* NCTC8325, and *Staphylococcus haemolyticus* ATCC14990) and *B. subtilis* revealed that within the same bacteria, interacting residues of DHp and Rec domains vary among different NarL TCSs (Fig. 5 and 6), which implies evolution of distinct specificities for cognate partners. Also interacting residues of orthologous TCSs were observed to be highly conserved, even across different strains and species of *Staphylococcus* (Fig. 5 and 6), highlighting the evolution of functional conservation.

The coevolution of interacting residues in cognate TCS pairs is fundamental to ensure specificity and reduces unwanted crosstalk with other TCS components (35). To gain further insight into the evolution of TCSs in maintaining signal fidelity between cognate HK-RR pairs, we analyzed the coevolution of residues between the Rec and DHp domains of cognate NarL TCSs. This was done by mutual information analysis of the amino acid alignments in cognate HKs and RRs of *S. aureus* (Fig. S7). In the *S. aureus* DesKR’s DHp and Rec domains, we did not identify a significant correlation between residues indicative of coevolution. This observation aligns with our expectations, given the low selection pressure on the DesKR HK and RR (Fig. 3; Table S4). Notably, among the interacting residues, a negative selection pressure was observed at the DHp residue R232_DesK_. This residue, conserved across NarL HK DHp domains (Fig. 6), forms a salt bridge with the *B. subtilis* D103_DesR_ (35).

**Fig 7:**
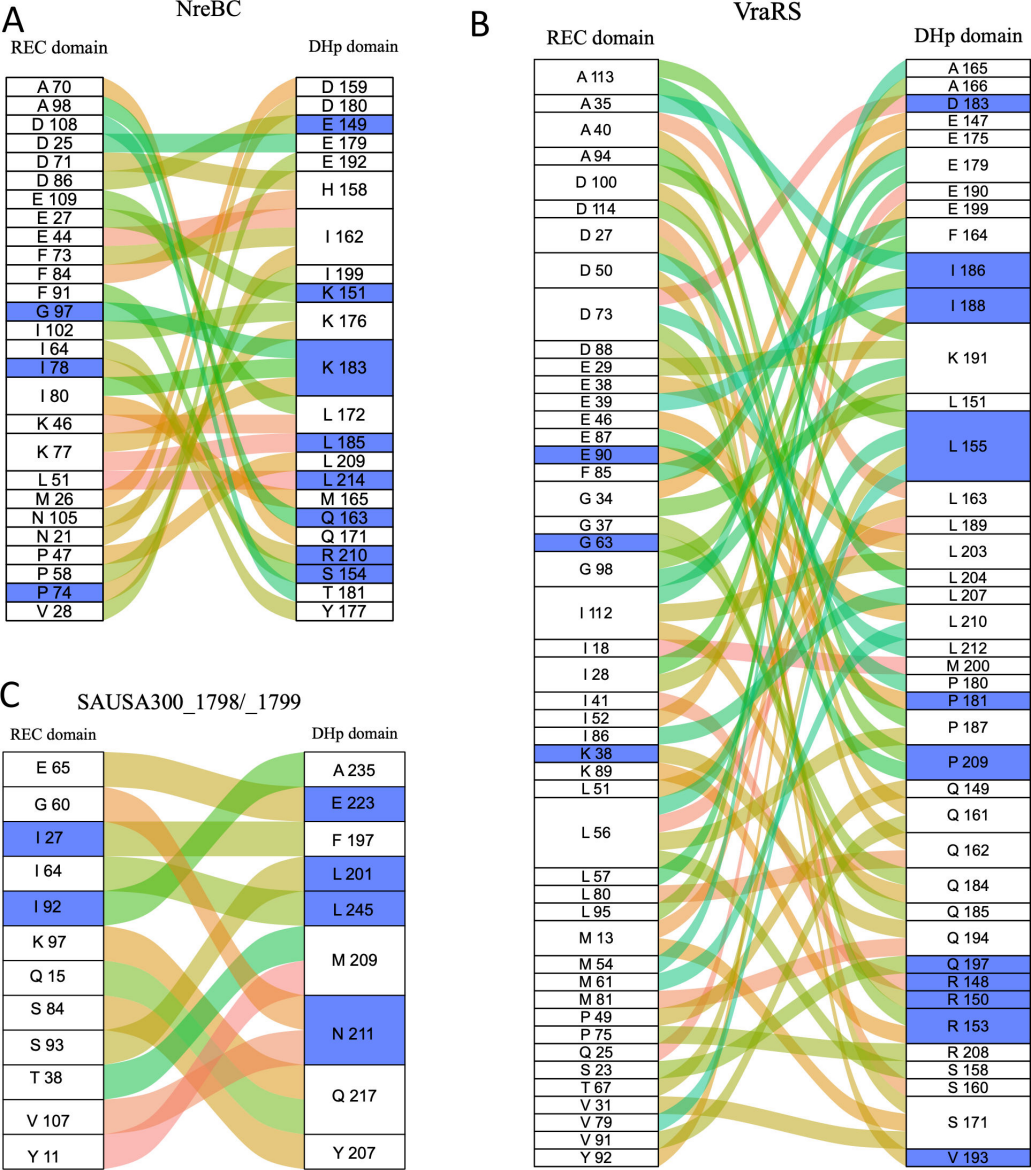
The coevolving residues of the Rec and DHp domain of the *S. aureus*** (A**) NreBC, (**B**) VraSR, and (**C**) SAUSA300_1798/1799. Curved lines show correlations between Rec and DHp residues. Blue shaded boxes represent residues that show evidence of posterior probability for negative selection. A threshold of 0.9 is set for inferring positive or negative selection.

Analysis of coevolutionary patterns in the DHp and Rec domains of NreBC highlighted a correlation between residues K176_(DHp)_ and N105_(Rec)_, both of which play a role in the interaction between these domains. Referring to the *B. subtilis* DesK-DesR cocrystal structure, K176_(DHp)_ establishes polar contacts with D108_(Rec)_ and E109_(Rec)_. In contrast, N105_(Rec)_ forms a salt bridge with R203_(DHp)_ (35). Additionally, D108_(Rec)_ and E109_(Rec)_ exhibit a strong correlation with T181_(DHp)_ and L172_(DHp)_, respectively. Furthermore, the residue Q163_(DHp)_, which creates van der Waals contacts within the RR groove, is highly correlated with A98_(Rec)_ (Fig. 7A). Among the coevolving residues identified, those from the DHp domain, including E149, K151, K183, L185, L214, Q163, R210, and S154, as well as those from the Rec domain, namely G97, I78, and P74, were deduced to be under negative selection pressure (Fig. 7A). Consistent with expectations, none of the residues that interact between the DHp and Rec domains exhibited positive selection pressure (Fig. 7A).

In the VraRS TCS, the interacting residues Q161_(DHp)_, F164_(DHp)_, S171_(DHp)_, and the I18_(Rec)_ were noted to coevolve with other residues in the cognate pair domains (Fig. 7B). Despite these coevolutionary tendencies, there was no evident coevolution between directly interacting residue pairs, and no selection pressures (neither positive nor negative) were identified for these residues or their coevolving counterparts (Fig. 7B).

In the SAUSA300_1798/1799 TCS, the residue F197 _(DHp)_, which forms van der Waals interactions with the RR groove, notably coevolves with I27_(Rec)_, which is under negative selection pressure. Intriguingly, the L201_(DHp)_ residue, aligning with the hydrophobic groove of the Rec domain, strongly correlates with S93_(Rec)_. Further emphasizing its evolutionary significance, L201_(DHp)_ exhibits evidence of negative selection pressure (Fig. 7C).

In general, the presence of negative selection pressures on various residues emphasizes their pivotal roles in system functionality and suggests evolutionary constraints to conserve these crucial interactions. Interestingly, while some TCSs showed discernible coevolution patterns, others did not, reflecting diverse evolutionary strategies or the influence of different environmental pressures.

## DISCUSSION

In this study focusing on *S. aureus* USA300, a predominant MRSA lineage, we unravel the complex interplay between the TCSs and uncover shared and unique virulence regulatory pathways and the genetic variations that mediate their signal specificity. Here, a comprehensive investigation of the virulence regulation of the TCSs was conducted using a transposon mutant library of the USA300 JE2 strain carrying inactivated HKs and RRs. We observed that some TCSs from the same family regulated different functions (Fig. 2; Fig. S2), which was expected due to their divergent functional evolution for sensing and responding to specific stimuli. Functional differences among TCS can be linked to the diversification of sensor and phosphotransfer domains in the HKs, where closely related TCS with similar domain architecture may show variations in specificity to cognate and non-cognate RRs (8, 18). Also, the modular architecture of the TCS, which includes signal sensing, phosphotransfer, and response, can contribute to variations in the impact of different TCSs on virulence determinants (8). Moreover, the integration of TCS within larger regulatory networks enables parallel or hierarchical regulation of virulence mechanisms or modulation by other active TCSs. For example, multiple interconnected TCSs such as ArlS, AgrAC, SrrAB, SaeRS, and LytT regulate biofilm formation (Fig. 2G).

This study comprehensively examined the TCSs present in *S. aureus* to delineate the virulence factors each TCS regulates. Our analysis was juxtaposed with existing literature that individually assessed the impact of various TCSs on virulence determinants with a predominant alignment between our findings and the established regulatory networks of TCSs, as documented in prior studies, particularly under diverse growth phases and media conditions. To address the discrepancies in TCS-mediated virulence regulation related to media conditions and bacterial growth phases, we standardized the profiling of virulence determinants by utilizing TSB as a general-purpose medium and implementing growth phase normalization in our assays. It seems plausible that certain TCS systems, like ArlSR, may influence the production of specific virulence factors, including nuclease production and RBC hemolysis. However, it is possible that the corresponding sensors were not activated under the tested conditions (Fig. 2I and J) (23, 58). Alternatively, the regulatory effects we observed could also result from cross-regulation among TCSs, shedding light on the broader regulatory network of these systems.

The experimental design used TCS transposon mutants to determine the direct effects of TCSs on various virulence traits. We selected transposon mutants with insertions that disrupt gene function, explicitly targeting those within the initial 60% of the gene sequence to ensure loss of function. To resolve inconsistencies arising from different virulence profiles among cognate pairs, we complemented selected TCS pairs to confirm their virulence phenotypes (Fig. S1D through F). Mostly, mutants in the HK or RR showed similar phenotypes during virulence profiling, thus internally validating each other. The interpretation is less straightforward for TCS mutant pairs with divergent phenotypic effects under selected conditions, e.g., cross-regulation between different TCSs like for the ArlRS, where nuclease production and RBC hemolysis were affected by the RR transposon mutant but not the HK (Fig. 2I and J; Fig. S1E and F) or a growth phase or media-dependent effect (22). Additionally, we compared the effects observed in the mutants with those in the WT strains to contextualize our findings within the framework of previously published research. This comparative analysis allowed us to corroborate the TCS-mediated virulence regulons reported in earlier studies and uncover additional TCSs that act as auxiliary regulatory systems for specific virulence determinants. Furthermore, the study contributes to understanding the complex regulatory roles of TCSs in *S. aureus*; by identifying both the confirmatory and novel elements of TCS-virulence regulation, we provide a more nuanced view of the pathogenic mechanisms at play, allowing future investigations into TCS functional dynamics.

Our study found that several TCSs exhibit similar regulatory profiles on virulence factor production despite belonging to different TCS families. This suggests divergent regulatory pathways have evolved despite similarities in HK and RR domains. Notably, global regulators AgrAC, SrrAB, and SaeRS displayed a high degree of correlation in their virulence profiles. The ArlRS and SaeRS TCSs indirectly influence each other’s activity through their connection with the Agr quorum-sensing system, which is strain dependent and affected by environmental conditions and the growth phase (4, 61, 62). The virulence profiles reveal an overlapping regulon between SaeRS and AgrAC, but SaeRS shares more common virulence profiles with SrrAB (Fig. S2). Both SaeRS and SrrAB belong to the OmpR family (Fig. 1).

We also identified cognate TCS pairs with similar virulence gene profiles, including DesKR, BraSR, PhoPR, NreBC, and YycHI, which can imply the evolution of specialized functions for these TCSs. Fascinatingly, the mutations of the HKs in the BraSR and GraSR displayed similarities in virulence profiles, which matches their similar domain architecture and sequence homology. However, their RR mutants exhibit distinct virulence profiles, suggesting functional divergence in RRs regulating downstream pathways.

Potential instances of independent functionality (e.g., AgrAC), crosstalk (e.g., ArlS-DesR and SrrB-VraR), and evolutionary divergence toward global regulon and specialized functionality (e.g., AgrAC and SaeRS) were observed among some of the TCSs. It is important to note that our assays used primary growth media and may lack the stimuli to activate TCS, resulting in less virulence factor production variation than previously published findings. For example, SrrAB exhibits both positive and negative regulation of protein A production under aerobic (Fig. 2F) and microaerobic conditions (48), respectively.

The TCS interconnections within broader regulatory networks enable parallel or hierarchical regulation of virulence mechanisms or modulation by the activity of other TCSs (22, 23, 63). For instance, multiple interconnected TCSs like ArlS, AgrAC, SrrAB, SaeRS, and LytTS regulate biofilm formation. This crosstalk among TCSs leads to complex and dynamic virulence factor regulation, impacting bacterial adaptation and pathogenicity. Crosstalk between TCS from the same family is expected due to common ancestry (5); however, it can also occur between different families (12). This depends on the evolution of specificity determinants mediating HK-DHp and RR-rec domain interactions for phosphotransfer. Given the selection pressure on TCS, continuous evolution could rewire signaling pathways or insulate TCS pathways through accumulated mutations in specificity determinants.

Previous studies conducted on *S. aureus* have demonstrated that mutants of the DesKR TCS exhibit higher tolerance to oxidative stress. However, these mutants also display increased autolysis, thinner cell walls, reduced antibiotic resistance, and lower pathogenicity *in vivo* (64). The role of DesKR in temperature adaptation and antibiotic resistance has been well established, particularly in terms of how temperature sensing affects its sensor-regulator interaction dynamics and physiological function (65, 66). Additionally, the identified DesKR in *S. aureus* functionally complements its well-characterized homolog in *B. subtilis* (59). This study used modeled structures of the NarL family TCS and the co-crystallized DesK-DesR complex from *B. subtilis* (35) to investigate the interacting residues between cognate TCS pairs. Sequence alignment revealed variations in these residues across different NarL TCSs from *S. aureus* strains and species. This highlighted the evolution of specificity determinants between cognate TCS pairs (8, 18), essential for reducing unwanted crosstalk and evolving new functions. We concurrently observed the conservation of specificity determinants within the same TCS across multiple strains. We also observed variations among related TCS within the same family from other bacterial species. While mutations in residues near interaction interfaces could influence specificity, we focused on the residues at the interaction interface between the HKs and RRs of *S. aureus* NarL TCS, referencing the DesK-DesR TCS from *B. subtilis*. Notably, we found species-specific variations in the interacting residues within the TCS belonging to the same family. This means that single amino acid changes in HK residues involved in specificity could lead to altered interactions with the RR as previously experimentally determined (8, 67, 68). Crucial similarities in biochemical properties, such as polarity and hydrophobicity, are revealed by variations in amino acids at the specificity determinants. These properties also result in similar interactions, such as hydrogen bonding (Fig. S5). This is expected, given the negative or neutral selection pressures across most sites in the DHp and Rec domains. With a few exceptions, such as DHp-aligned residues K245 (hydrophilic) and Y245 (hydrophobic depending on context and modification) and Rec D114 (hydrophilic), variants in all interacting residues in both domains are conserved in terms of hydrophobicity and hydrophilicity.

The observed high negative selection across the TCS sites indicates genetic constraints that shape their evolution in bacteria. This implies that mutations disrupting TCS gene function are generally harmful and likely eliminated by natural selection. As a result, the maintenance of new kinase-regulator pairs in the genome depends on intermediate steps between the original and new pairs being neutral or negative, allowing for the gradual evolution of new pairs without disrupting TCS system function. High negative selection pressures also indicate strong evolutionary constraints on these genes, potentially limiting bacterial adaptability to new environments or response to changing conditions. Considering the importance of TCS genes for bacterial survival and fitness, mutations disrupting their function are likely strongly selected against since they may negatively affect the ability of the bacteria to adapt to changing environmental conditions.

Amino acid coevolution and selection pressure analysis are fundamental for examining the specificity and molecular recognition in various HK-RR interactions (8, 67). From the coevolution analysis, the interacting residues of the cognate HK and RR do not consistently exhibit a strong correlation (Fig. 7). We hypothesize that this may be attributed to the selection cutoff for the coevolving residues in the Rec and DHp domain, which serves to minimize the coevolutionary signals between the interacting residues and reduces noise in the mutual information analysis. Our findings do indicate a strong coevolutionary relationship between certain adjacent residues and one or more of the interacting pairs. This may suggest that residues adjacent to the interacting residues might influence the interaction between the two proteins by indirectly affecting the interface by modulating the conformation, stability, or orientation of the interacting residues. The negative selection pressures on certain residues indicate that evolution preserves these crucial interactions. As such, some TCS residues in the DHp and Rec exhibit coevolution patterns, while others do not, suggesting different evolutionary strategies.

Mutations in specificity residues require corresponding changes in their cognate regulators to maintain each pathway’s operation and avoid crosstalk as they diverge (13, 37). Identifying specific residues that contribute to this specificity is crucial, as TCS signaling pathways are not entirely insulated, as evidenced by the virulence regulation and global regulons. While this study focused on the overlapping regulons due to specificity at the phosphotransfer level DHp and Rec domains, other determinants include interactions of the effector-binding domain to specific motifs, receptor dimerization, or the response to specific environmental signals by the sensor kinase (8, 18).

In this study, using multiple sequence alignments in conjunction with the cocrystal structure of the NarL DesK-DesR complex helped identify specific residues underlying phosphotransfer specificity between HKs and RRs. As genome sequence databases expand, covariation analysis has proven helpful in the absence of high-resolution structures of complex cognate pairs. However, they cannot definitively identify the critical amino acid residues for specificity or reveal the degree to which each residue contributes to substrate selection. Experimental evidence such as alanine scanning can provide further confirmation of specificity determinants in TCS.

In conclusion, an exhaustive exploration of signal fidelity determinants in TCS and their implications on virulence factor regulation in bacteria enriches our comprehension of bacterial evolution, adaptation, and the mechanisms governing their pathogenicity. This serves as a foundation for advancing synthetic biology through the manipulation of bacterial regulatory networks and rewiring their modularity.

Considering the growing interest in targeting TCSs for anti-virulence strategies, a comprehensive understanding of their function, interactions, and specificity becomes paramount. This knowledge will not only provide insights into bacterial physiology but also inform more effective therapeutic interventions.

## SUPPLEMENTAL MATERIAL

Supplemental figures msystems.00130-24-s0001.pdf Fig. S1 to S7.

Table S1 msystems.00130-24-s0002.xlsx Gene names of the *S. aureus* TCSs HKs and RRs.

Table S2 msystems.00130-24-s0003.xlsx Strains.

Table S3 msystems.00130-24-s0004.xlsx Primers.

Table S4 msystems.00130-24-s0005.xlsx Negative and positive selection pressure per site of the *S. aureus* TCS HKs and RRs.

ASM does not own the copyrights to Supplemental Material that may be linked to, or accessed through, an article. The authors have granted ASM a non-exclusive, world-wide license to publish the Supplemental Material files. Please contact the corresponding author directly for reuse.
