# Supplementary material for: Comprehensive virulence profiling and evolutionary analysis of specificity determinants in Staphylococcus aureus two-component systems
Source: mSystems. 2024 Mar 12;9(4):e00130-24. doi: 10.1128/msystems.00130-24 (PMC11019936; doi:10.1128/msystems.00130-24)
Supplement: Supplemental figures — Fig. S1 to S7. [file msystems.00130-24-s0001.pdf]

Supplementary Material

Title: Comprehensive Virulence Profiling and Evolutionary Analysis of Specificity Determinants in *Staphylococcus aureus* Two-Component Systems

Authors: Stephen Dela Ahator[1*], Karoline Wenzl[1], Kristin Hegstad[1,2], Christian S. Lentz[1], Mona Johannessen[1*]

Affiliation:

[1] Centre for New Antibacterial Strategies (CANS) and Research Group for Host-Microbe Interactions, Department of Medical Biology, Faculty of Health Sciences, UiT- The Arctic University of Norway, Tromsø, Norway

[2] Norwegian National Advisory Unit on Detection of Antimicrobial Resistance, Department of Microbiology and Infection Control, University Hospital of North Norway, Tromsø, Norway

* Corresponding author: Mona Johannessen and Stephen Dela Ahator

Fig S1

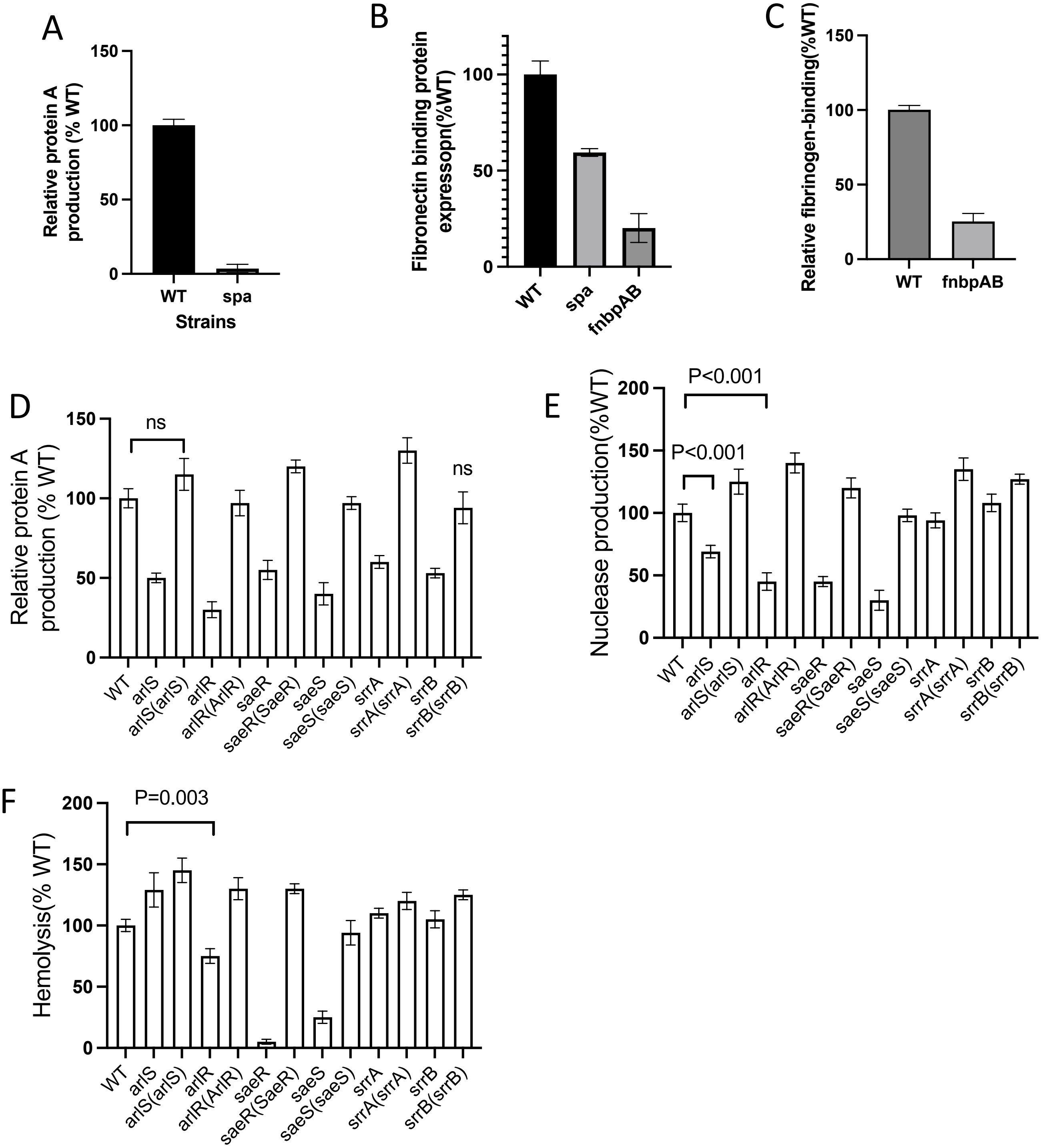

A) The levels of protein A production in strain spa relative to the WT strain. **B**) Fibronectin-Binding Proteins, and **C**) Fibrinogen-binding proteins in *S. aureus*. The levels of these virulence determinants in the protein A null mutant (*spa*) and the double mutant for fibronectin-binding proteins A and B (*fnbpAB*), also implicated in fibrinogen binding were compared to the WT, with values normalized to 100%. The presented data depict the mean ± SD from four independent experiments. **D**) Relative production of protein A in the transposon HK and RR mutants of ArlSR, SaeSR, SrrAB TCS and their in-trans complements. The protein A production represents the mean ± SD of 3 independent experiments compared to the normalised 100% values of the WT. **E** and **F** represent the relative nuclease and hemolysis production respectively in the transposon HK and RR mutants of ArlSR, SaeSR, SrrAB TCS and their in-trans complements. The level of hemolysis and nuclease production were compared to the WT level normalised at 100% and represents the mean ± SD of 6 independent experiments. P value < 0.05 is considered significant and P value > 0.05 is considered non-significant (ns)

Fig S2

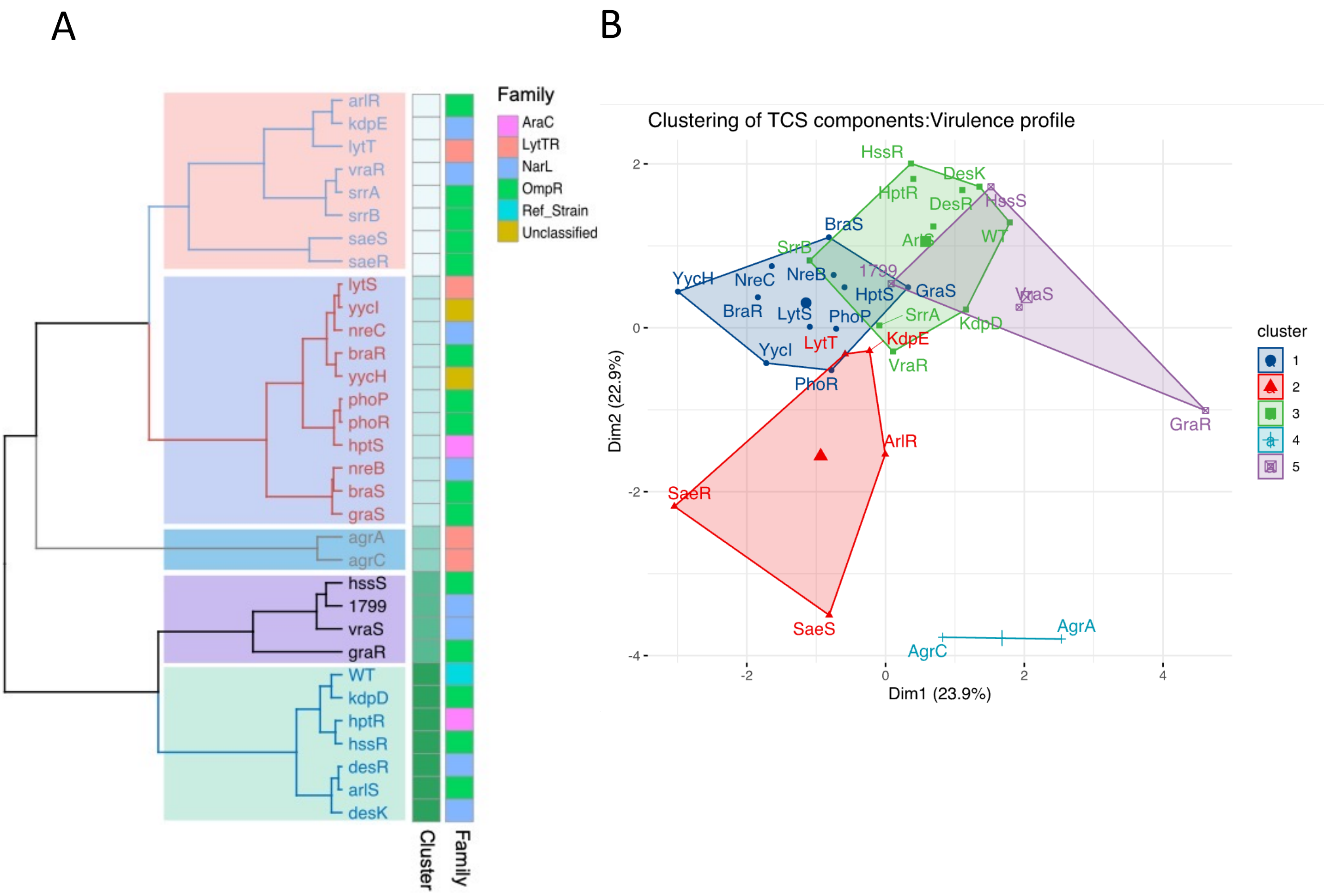

A) Cluster-based analysis of the virulence profiles of the HKs and RRs of TCS in *S. aureus* USA300_FPR3757. The dendrogram shows clusters based on Euclidean distance, with each component annotated according to its respective family. **B**) PCA analysis of the virulence profiles of the TCS components. Clusters were determined using the variance explained by the PCA analysis (Dim5: 82.5%).

Figure S3

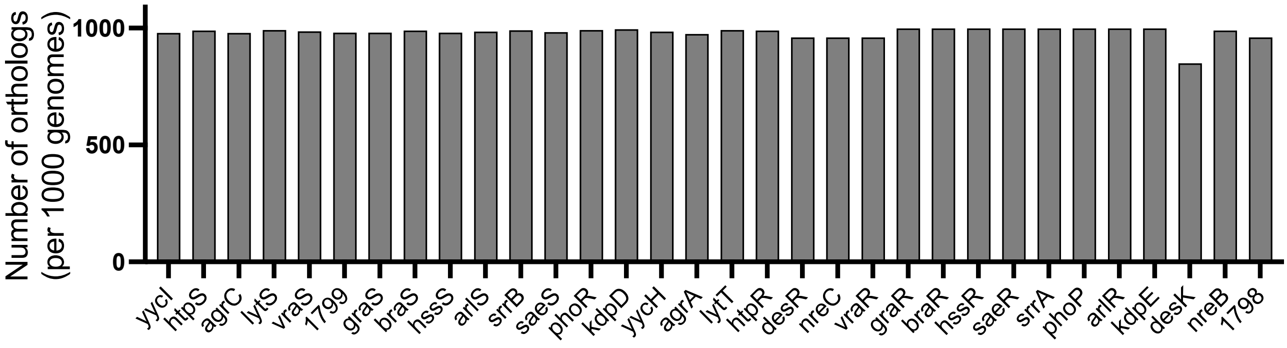

Distribution of TCS component orthologs in a 1000 genomes of *S. aureus* obtained from NCBI Datasets (from the year 2000-2022). Orthologs were identified using the Orthofinder software with the TCS amino acid sequences from *S. aureus* USA300_FPR3757 as reference.

Figure S4

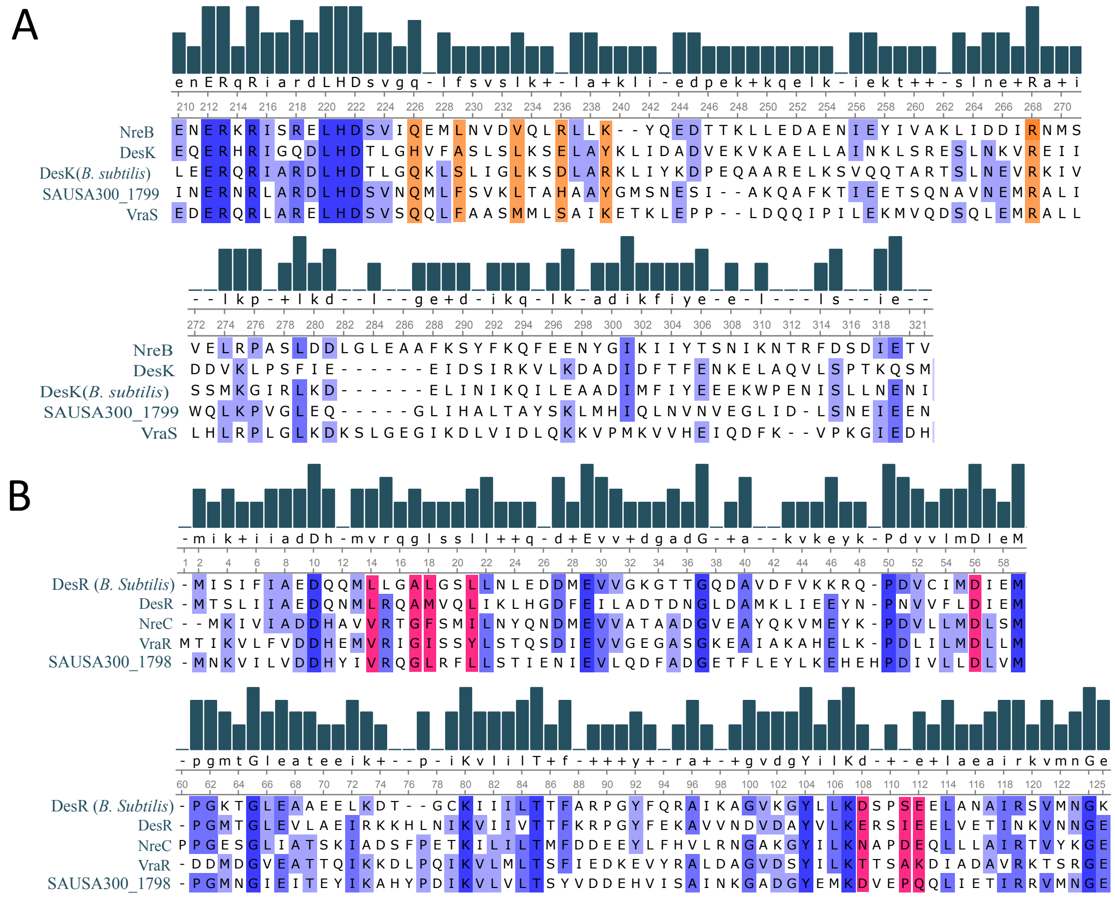

(**A**) The sequence alignment of the DHp domain of the *S. aureus*_USA300 HKs NreB, DesK, SAUSA300_1799, and VraS with the DHp domain from the *B. subtilis* DesK. The conserved residues are highlighted in shade of blue from lowest to highest. The consensus are shown above the residue position. Orange shades represent the aligned residues with experimental evidence of interaction between the DHp and the cognate Rec domains of the *B. subtilis* DesK-DesR TCS.

(**B**) The sequence alignment of the Rec domain of the *S. aureus*_USA300 RRs, DesR, NreC, VraR, and SAUSA300_1798, with the Rec domain from the *B. subtilis* DesR. The conserved residues are highlighted in shade of blue from lowest to highest. The consensus are shown above the residue position with the pink shade representing the residues that align with experimentally determined interactions between the Rec and the cognate DHp domains (**A**) of the *B. subtilis* DesK-DesR TCS.

Figure S5

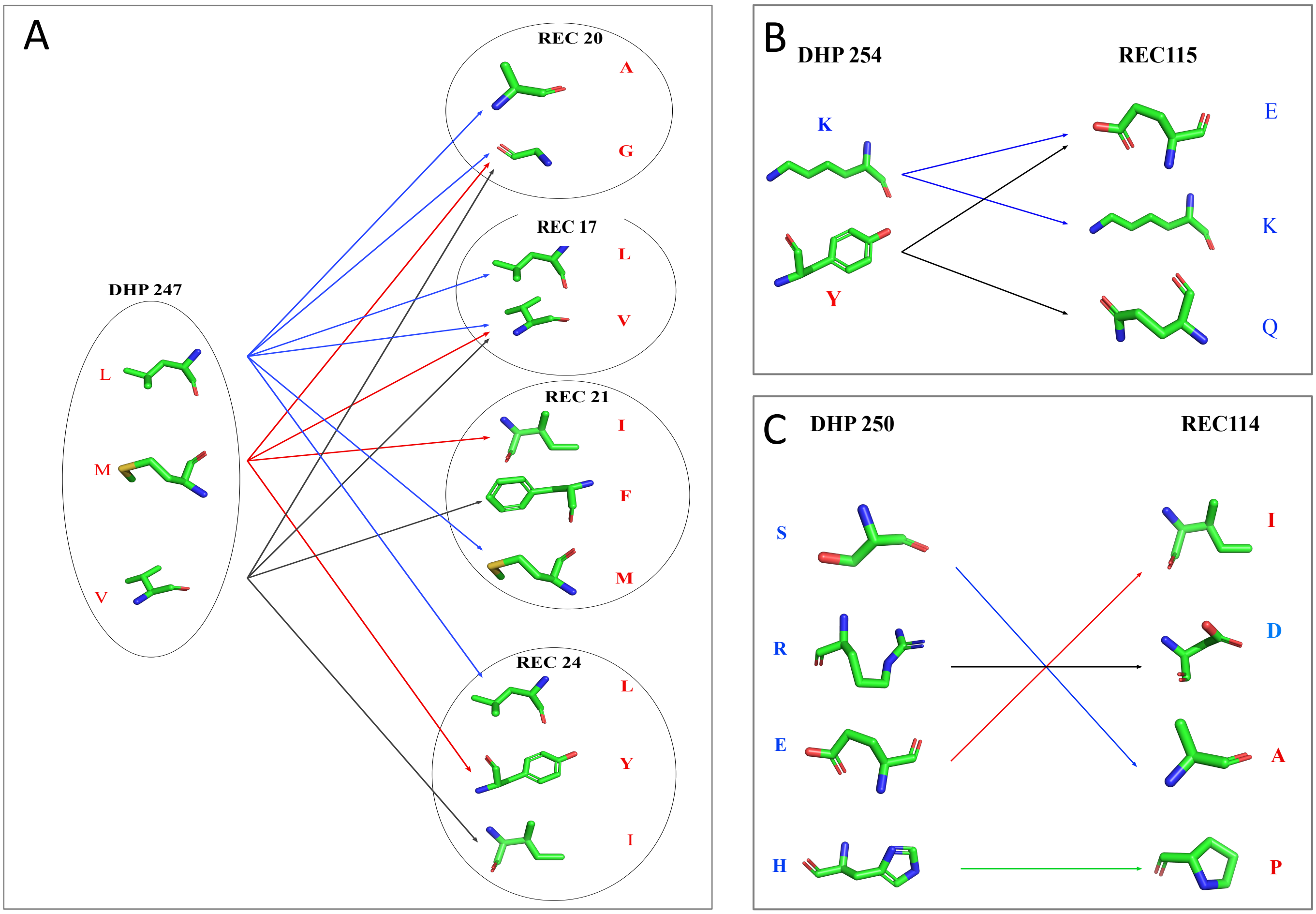

Fig S5. Variations in the interacting residues between the DHp and Rec domains of the *S. aureus* NarL family TCS HKs and RRs: **A**) Displays the amino acid variations in the DHp domain that interact with residues around the hydrophobic groove in the Rec domain. **B**) Shows variations in the interacting residues $254_{(DHp)}$ and $115_{(Rec)}$. **C**) Illustrates variations in the interacting residues $250_{(DHp)}$ and $114_{(Rec)}$. Amino acid positions are based on the sequence alignment from the HKs and RRs from the Staphylococcus species and B. subtilis sequence alignments shown in Fig 5 and Fig 6. Red-labeled amino acids are hydrophobic, while blue-labeled amino acids are hydrophilic. Arrows indicate the interacting pairs based on the cognate DHp-Rec pairs.

Fig S6

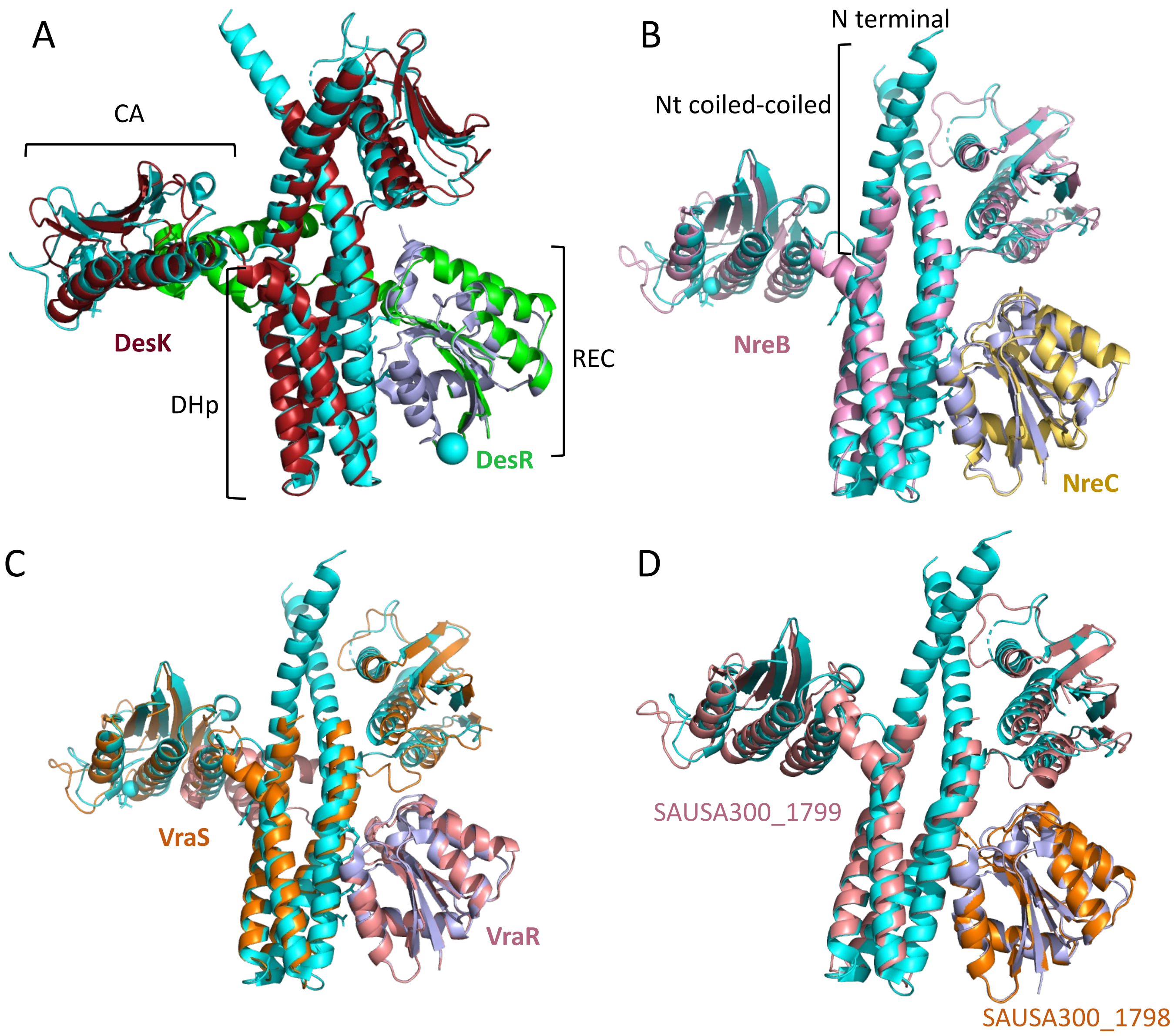

The molecules colored cyan and light blue are 5IUJ HK and RR of the *B. subtilis* DesK-DesR.

(**A**) The deep red and green are the HK and RR of the DesK and DesR of *S. aureus* USA300. Two molecules of the RR are known to bind a dimer of the HK; however, for clarity, only one molecule of RR is shown for the DesK-DesR complex. The *S. aureus* DesK residue superposition onto the HK of the DesK-DesR cocrystalised complex (5IUJ) revealed a very similar structure with 1.993 Å rsmd aligning 2296 atoms. The S. aureus DesR residue superimposed onto the RR of the model 5IUJ with an rsmd of 0.351 Å aligning 626 atoms. The superposition of the DesK HKs from both *S. aureus* and *B. subtilis* revealed a similar structure aside from the coiled-coil region.

(**B**) The light pink and yellow colored cartoon structures are the HK and RR of the SAUSA300_1799 and SAUSA300_1798. Only one molecule of the SAUSA300_1798 is shown in both RRs of the superimposed complex. The SAUSA300_1799 residues superposition onto the HK of the DesK-DesR cocrystalised complex (5IUJ) revealed a very similar structure with 2.098 Å rsmd aligning 2157 atoms. The *S. aureus* SAUSA300_1798 residue superimposed onto the RR of the model 5IUJ with an rsmd of 1.096Å aligning 634 atoms.

(**C**) The orange and salmon-colored cartoon structures are the HK and RR of the VraS and VraR. The VraS residues superposition onto the HK of the DesK-DesR cocrystalised complex (5IUJ) revealed a 2.487 Å rsmd aligning 2006 atoms. The VraR residue superimposed onto the RR of the model 5IUJ with an rsmd of 0.322 Å aligning 577 atoms.

(**D**) The salmon and orange colored cartoon structures are the HK and RR of the NreB and NreC. The NreB residues superposition onto the HK of the DesK-DesR cocrystalised complex (5IUJ) revealed a 2.114 Å rsmd aligning 1959 atoms. The VraR residue superimposed onto the RR of the model 5IUJ with an rsmd of 0.402 Å aligning 601 atoms.

Fig S7

A

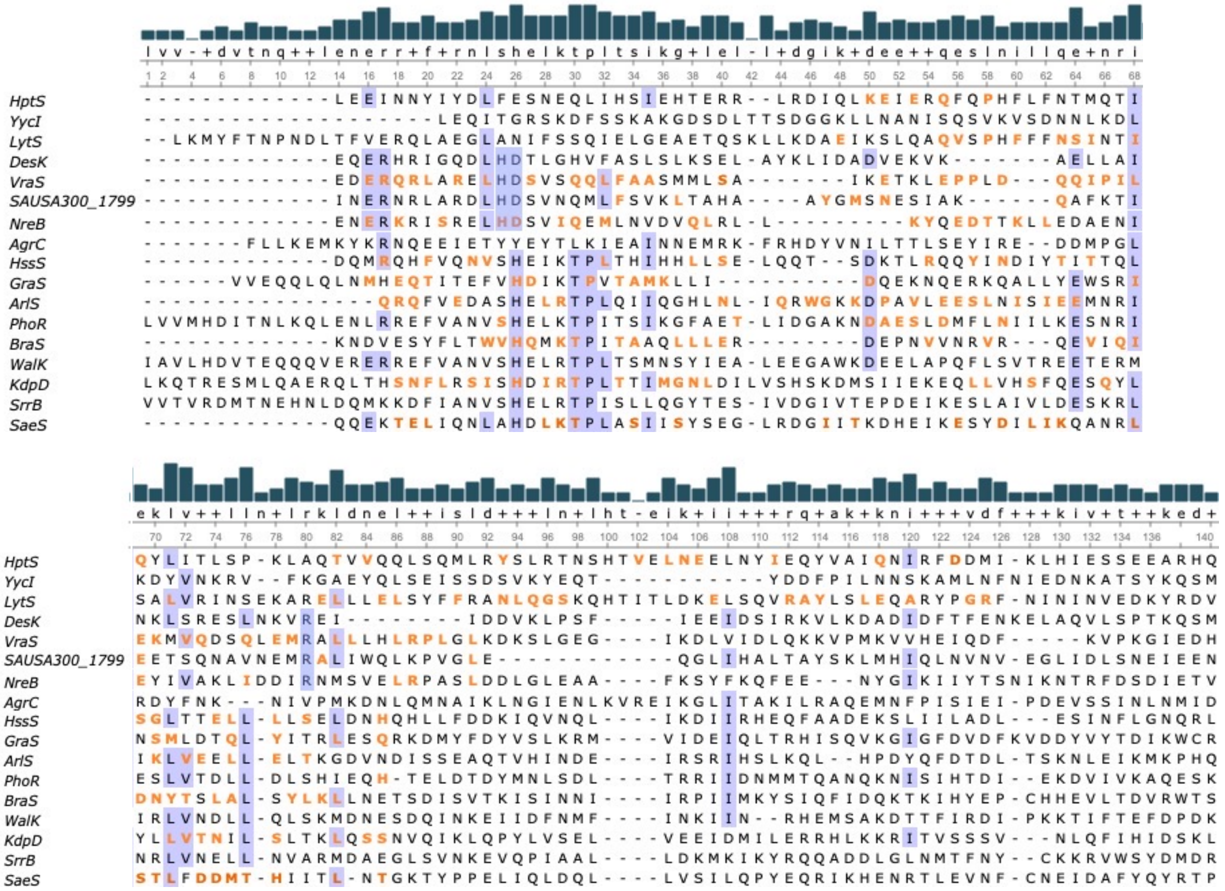
HptS
YycI
LytS
DesK
VraS
SAUSA300_1799
NreB
AgrC
HssS
GraS
ArlS
PhoR
BraS
WalK
KdpD
SrrB
SaeS

B

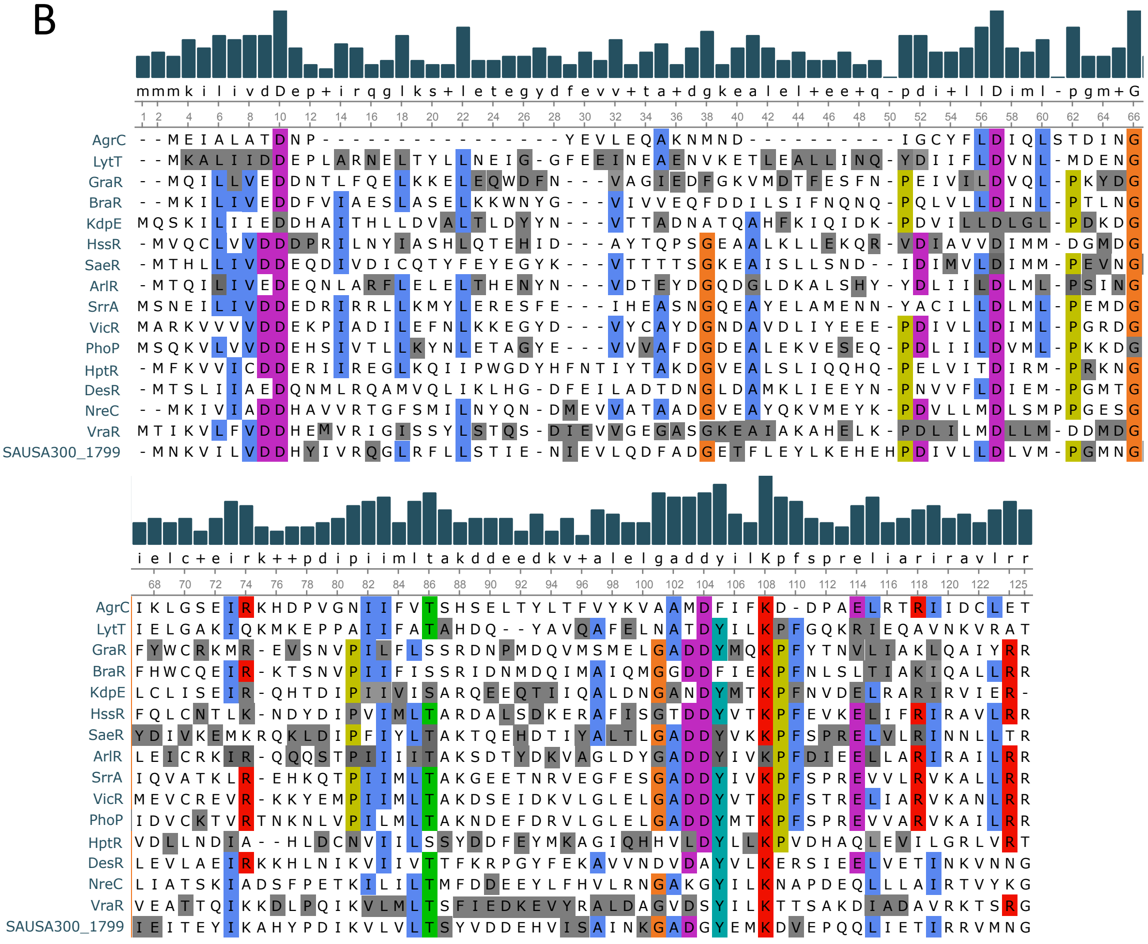
AgrC
LytT
GraR
BraR
KdpE
HssR
SaeR
ArlR
SrrA
VicR
PhoP
HptR
DesR
NreC
VraR
SAUSA300_1799

Fig S7:

A) The sequence alignment of the DHp domain of the TCS HKs in the *S. aureus*_USA300. The orange font colored residues represent the selected coevolving amino acids in the DHp domain with residues in the RR rec domain. The blue vertical highlighted residues show the conserved residues between the DHp domains. The consensus is shown above the residue position. Gaps are introduced to maximize alignment are indicated by the dashes.

B) The sequence alignment of the Rec domain of the TCS RRs in the *S. aureus*_USA300. The grey-shaded residues represent the selected coevolving amino acids in the rec domain with residues in the HK DHp domain in **A**. The consensus is shown above the residue position. Gaps are introduced to maximize alignment are indicated by the dashes.
